# Mitigation of endemic GI-tract pathogen-mediated inflammation through development of multimodal treatment regimen and its impact on SIV acquisition in rhesus macaques

**DOI:** 10.1371/journal.ppat.1009565

**Published:** 2021-05-10

**Authors:** Rachele M. Bochart, Kathleen Busman-Sahay, Stephen Bondoc, David W. Morrow, Alexandra M. Ortiz, Christine M. Fennessey, Miranda B. Fischer, Oriene Shiel, Tonya Swanson, Christine M. Shriver-Munsch, Hugh B. Crank, Kimberly M. Armantrout, Aaron M. Barber-Axthelm, Charlotte Langner, Cassandra R. Moats, Caralyn S. Labriola, Rhonda MacAllister, Michael K. Axthelm, Jason M. Brenchley, Brandon F. Keele, Jacob D. Estes, Scott G. Hansen, Jeremy V. Smedley

**Affiliations:** 1 Infectious Disease Resource, Oregon National Primate Research Center, Oregon Health and Science University, Beaverton, Oregon, United States of America; 2 Division of Pathobiology and Immunology, Oregon National Primate Research Center, and Vaccine and Gene Therapy Institute, Oregon Health and Science University, Beaverton, Oregon, United States of America; 3 Barrier Immunity Section, Lab of Viral Diseases, National Institute of Allergy and Infectious Diseases, National Institutes of Health, Bethesda, Maryland, United State of America; 4 AIDS and Cancer Virus Program, Frederick National Laboratory for Cancer Research, Frederick, Maryland, United States of America; 5 Division of Comparative Medicine, Oregon National Primate Research Center, Oregon Health and Science University, Beaverton, Oregon, United States of America; University of Minnesota, UNITED STATES

## Abstract

Here, we assessed the efficacy of a short-course multimodal therapy (enrofloxacin, azithromycin, fenbendazole, and paromomycin) to eliminate common macaque endemic pathogens (EPs) and evaluated its impact on gastrointestinal (GI) microbiota, mucosal integrity, and local and systemic inflammation in sixteen clinically healthy macaques. Treatment combined with expanded practices resulted in successful maintenance of rhesus macaques (RM) free of common EPs, with no evidence of overt microbiota diversity loss or dysbiosis and instead resulted in a more defined luminal microbiota across study subjects. Creation of a GI pathogen free (GPF) status resulted in improved colonic mucosal barrier function (histologically, reduced colonic MPO+, and reduced pan-bacterial 16s rRNA in the MLN), reduced local and systemic innate and adaptive inflammation with reduction of colonic Mx1 and pSTAT1, decreased intermediate (CD14+CD16+) and non-classical monocytes (CD14-CD16+), reduced populations of peripheral dendritic cells, Ki-67+ and CD38+ CD4+ T cells, Ki-67+IgG+, and Ki-67+IgD+ B cells indicating lower levels of background inflammation in the distal descending colon, draining mesenteric lymph nodes, and systemically in peripheral blood, spleen, and axillary lymph nodes. A more controlled rate of viral acquisition resulted when untreated and treated macaques were challenged by low dose intrarectal SIVmac239x, with an ~100 fold increase in dose required to infect 50% (AID_50_) of the animals receiving treatment compared to untreated controls. Reduction in and increased consistency of number of transmitted founder variants resulting from challenge seen in the proof of concept study directly correlated with post-treatment GPF animal’s improved barrier function and reduction of key target cell populations (Ki-67+ CD4+T cells) at the site of viral acquisition in the follow up study. These data demonstrate that a therapeutic and operational strategy can successfully eliminate varying background levels of EPs and their associated aberrant immunomodulatory effects within a captive macaque cohort, leading to a more consistent, better defined and reproducible research model.

## Author summary

Simian Immunodeficiency virus in macaques remains the most translational model for HIV. Macaques are also key models for other infectious diseases and colitis, where background colon health and inflammation could confound experimental results. To address these issues SPF breeding colonies were established to exclude specific viruses detrimental to mucosal and systemic health. To further improve macaque models, we established a gastrointestinal pathogen free (GPF) colony by administration of a multimodal therapeutic regimen for common endemic pathogens (EPs) and established expanded operational practices for continued exclusion. Through extensive longitudinal microbiota, parasitology, microbiology, and tissue sampling we demonstrated that our treatment and exclusion practices successfully eliminated common EPs, improved mucosal barriers, and reduced mucosal and systemic inflammation resulting in less inter-animal variation without overt evidence of microbiota disruption. Finally, compared to treatment-naïve controls, GPF animals challenged with SIV intrarectally demonstrated a more controlled and consistent rate of SIV acquisition, further indicating underlying EPs even at subclinical levels may cause deleterious variations between study subjects. Collectively the GPF macaque represents a model that eliminates a significant source of morbidity, reduces inter-animal variability, and thus has the potential to improve both animal welfare and research outcomes.

## Introduction

Nonhuman primate (NHP) models of infectious disease, immunology, and inflammation are among the most predictive for human outcomes due to their close genetic and physiologic similarities. However, nosocomial viral, bacterial, and parasitic endemic pathogens (EPs) can have profound negative impacts on these research models due to immunologic and physiological changes induced by these infections. In order to control for viral EPs in captive NHP colonies, institutions have established specific pathogen free (SPF) and expanded specific pathogen free (eSPF) rhesus macaque colonies, excluding four and nine viruses, respectively. SPF colonies exclude viruses such as *Macacine herpesvirus* 1, simian retrovirus type D, and simian immunodeficiency virus, all of which can have a significant impact on the macaque immune system and overall health, as well as present a zoonotic risk to workers[1]. Exclusion of these agents allows for a safer, more controlled, and well-defined biomedical research model [1]. However, these viruses represent only a small proportion of EPs which affect macaque health and introduce variability into research models.

Common bacterial and parasitic EPs within captive macaque populations include the gastrointestinal (GI) microbes *Campylobacter* spp. *(C*. *coli and C*. *jejuni)*, *Shigella* spp., *Balantidium* spp., *Entamoeba* spp., *Giardia* spp., *Strongyloides* spp., and *Trichuris* spp. [2]. These EPs have wide inter-institutional prevalence and can create significant mucosal inflammation concomitant with GI barrier disruption [2,3]. As has been shown with dextran sulfate sodium (DSS) treatment, disruptions of the mucosal barrier and the associated colitis can result in significant local and systemic inflammatory changes that mimic key pathologic features of simian immunodeficiency virus (SIV), inflammatory bowel disease, and ulcerative colitis [4,5]. Despite the importance of GI barrier integrity on local and systemic inflammation and the potential impact this can have on enhancing acquisition in low-dose SIV rectal transmission studies, these pathogens are often present at variable and sometimes high prevalence in rhesus macaques resulting in clinical and subclinical disease [6] at different times in different animals during the course of a study, which can serve as a confounding variable.

We first conducted a proof of concept study to evaluate the importance of eliminating common bacterial and parasitic endemic pathogens on SIV acquisition. There was an ~100 fold increase in intrarectal (IR) SIVmac239X dose required to infect 50% of the animals (AID_50_) receiving treatment for common GI pathogens utilizing a short course multimodal therapeutic regimen (enrofloxacin, paromomycin, and fenbendazole), compared to untreated animals. We then sought to create and evaluate a “gastrointestinal pathogen free” (GPF) cohort of animals by utilizing a short course multimodal therapeutic regimen (enrofloxacin, paromomycin, fenbendazole, and azithromycin (added to target the elimination of *Campylobacter* spp.)). designed to eliminate common enteric EPs prior to study initiation. Towards that end, we assessed known pathogenic bacteria and parasites before and after treatment based on fecal culture and parasitology results, as well as the impacts of the treatment on the colonic microbiota. Furthermore, we evaluated inflammatory and mucosal barrier changes before and after achieving GPF status using histology, immunohistochemistry (IHC), pan-bacterial 16s rRNA RNAscope, and flow cytometry techniques within the GI-tract and GI-proximal sites (i.e., mesenteric lymph nodes and liver), as well as distal sites (i.e., spleen, blood, bronchoalveolar lavage, bone marrow, and axillary lymph nodes). Finally, we established expanded operational procedures to ensure maintenance of GPF status, and documented their efficacy maintaining this status through serial microbiological and parasitological examinations. We hypothesized that the administration of the multimodal treatment regimen and implementing refined exclusion practices would allow for maintenance of clinically healthy macaques free of common EPs, as well as improve the integrity of the gastrointestinal mucosa resulting in overt immunological and microbiota stabilization across study subjects, thus creating a more defined macaque research model.

Our study demonstrated that the short course multimodal treatment improved GI barrier function, reduced local and systemic inflammation in both the innate and adaptive immune systems, and reduced inter-animal variability. We also demonstrated that the regimen and associated practices were capable of creating and maintaining GPF status in rhesus macaques for over 6 months. Finally, the regimen did not result in the type of Proteobacterial bloom that would be of concern with antibiotic treatment, but resulted in a microbiome enriched for beneficial taxa including *Clostridium* clusters IV and XIVa, and free of pathogenic *Shigella* or *Campylobacter* spp. Together these findings indicate the potential to reduce the effects of bacterial and parasitic EPs on rhesus macaque models where variability in the timing and amount of associated clinical and subclinical disease, impacts to mucosal barrier function, and associated inflammation could significantly impact experimental outcomes.

## Results

### Proof of concept study evaluating acquisition of IR SIVmac239X and enumeration of transmitted/founder (T/F) variants in multimodal therapy treated and untreated rhesus macaques

IR challenge of untreated macaques with 1000 IU and 3000 IU resulted in infection of 1 out of 2 and 2 of 2 animals, respectively (Table 1). The number of detectable T/F variants for the 1000 IU dose infected animal was 2 while the 3000 IU animals were infected with 6 and 8 variants. Based on both infection events and the number of T/F variants, we estimate an AID_50_ of ~1000 IU for rectal challenge with this stock. Of six animals that received the multimodal treatment regimen (enrofloxacin, fenbendazole, and paromomycin) and maintained in a facility where all animals had been treated and testing demonstrated exclusion of common protozoan and metazoan parasites and *Shigella* spp. prior to challenge, none had detectable virus when identically dosed with 3000 IU. We then increased the dose to 30,000 IU which resulted in infection of 1 of 4 animals, with 2 T/F variants in the infected animal. We therefore increased the dose to 300,000 IU which resulted in infection of 6/6 animals with 1–4 T/F per animal as shown in Table 1. The animals that received the multimodal treatment regimen had an estimated AID_50_ of ~100,000 IU, a 100-fold increase over the animals that did not receive the regimen. As the primary goal of this experiment was to determine the appropriate IR challenge dose to achieve a 100% rate of infection with consistent numbers of T/F variants, appropriate samples were not obtained to evaluate the EP status, nor the inflammatory, mucosal barrier or microbiome impacts of the multimodal therapy. We therefore conducted a follow-up study to evaluate the impacts on these key parameters in order to determine the changes that were associated with this drastic change in viral acquisition.

**Table 1:** Evaluation of acquisition of intrarectal challenged SIVmac239X and enumeration of transmitted/found variants in multimodal treated and untreated rhesus macaques.

Multimodal therapy naive				
Animal	Dose	Infection	T/F counts	AID50 ~1,000 IU
ZD47	1,000 IU	No	0	
DD36	1,000 IU	Yes	2	
			
ZD49	3,000 IU	Yes	6	
ZD76	3,000 IU	Yes	8	
Multimodal therapy treated				
Animal	Dose	Infection	T/F counts	
4470	3,000 IU	No	0	AID50 ~100,000 IU
4402	3,000 IU	No	0	
4376	3,000 IU	No	0	
4399	3,000 IU	No	0	
BF61	3,000 IU	No	0	
CJ2F	3,000 IU	No	0	
4550	30,000 IU	No	0	
4317	30,000 IU	No	0	
4324	30,000 IU	No	0	
4543	30,000 IU	Yes	2	
DEJ8	300,000 IU	Yes	1	
4397	300,000 IU	Yes	2	
DEZ2	300,000 IU	Yes	2	
KNL	300,000 IU	Yes	2	
4317	300,000 IU	Yes	2	
4324	300,000 IU	Yes	4	

### Evaluation of the multimodal therapy regimen in rhesus macaques

Sixteen male rhesus macaques were used to assess the levels of inflammation at gastrointestinal, draining and systemic sites, as well as colonic mucosal integrity following a short course multimodal treatment aimed at establishing a GPF macaque model. Comprehensive sampling inclusive of peripheral blood, axillary and mesenteric lymph nodes (ALN and MLN), spleen, liver, distal descending colonic mucosa, and fecal samples were obtained prior to and after a short course multimodal treatment (enrofloxacin, azithromycin, paromomycin, and fenbendazole) regimen. Distal descending colonic mucosa samples were used to assess local inflammation and barrier integrity via IHC and flow cytometry at the site where a typical rectal SIV challenge would occur. Lymphoid tissues, liver, and blood were utilized to assess local GI-tract draining sites (liver and MLN) via flow cytometry and IHC, and systemic responses (blood, spleen, scopeless bronchoalveolar lavage (BAL), bone marrow (BM), and ALN) via flow cytometry. Fecal samples were utilized for parasitology, microbiology, and microbiome analyses; longitudinal microbiology and parasitic examinations continued to assess initial efficacy of treatment and the effectiveness of expanded EP exclusion practices to maintain this status. Baseline microbiology cultures and parasitic examinations identified ten of sixteen animals with established EPs (*Campylobacter coli* (9/10), *Shigella flexneri* (1/10), and *Trichuris* spp. (6/10)) (Table 2). No animals developed significant clinical GI disease requiring treatment during the 6-month evaluation period after establishment of GPF status.

**Table 2:** Pre-treatment regimen fecal microbiology culture and parasitology examination results.

Animal	Microbiology	Parasitology
RM1	N	-
RM2	N	*Trichuris* spp.
RM3	N	-
RM4	N	-
RM5	N	-
RM6	*Campylobacter coli*	-
RM7	*Campylobacter coli*	*Trichuris* spp.
RM8	*Campylobacter coli*	*Trichuris* spp.
RM9	*Campylobacter coli*	*Trichuris*
RM10	N	-
RM11	N	-
RM12	*Campylobacter coli*	*Trichuris* spp.
RM13	*Campylobacter coli*	-
RM14	*Campylobacter coli*	-
RM15	*Shigella flexneri*, *Campylobacter coli*	*Trichuris* spp.
RM16	*Campylobacter coli*	-

(-) Negative, (N) Normal enteric flora

### Short course multimodal treatment regimen results in a consistent fecal microbiome across study animals

The short course multimodal treatment regimen employed drugs capable of eliminating the common GI bacterial and parasitic infections seen in rhesus macaques. Fenbendazole and paromomycin are effective against the common metazoan and protozoan parasites [7,8]. Enrofloxacin is highly efficacious against *Shigella* spp. [9], and azithromycin (added to the regimen for the ONPRC animals given the high prevalence of *Campylobacter* spp. in this colony) is similarly efficacious against *Campylobacter* spp. [10]. Doses and durations were based on veterinary experience with the drugs and agents being treated. The choice to use enrofloxacin, paromomycin, and fenbendazole concurrently was based on the bactericidal activity of these drugs and prior experience eliminating the common pathogens (except *Campylobacter* spp.) using this combined regimen, as well as it was utilized for the proof-of concept study cohort. The decision to administer azithromycin (in an attempt to eliminate *Campylobacter* spp.) after completion of the other drug regimens was rooted in concerns about the potential of azithromycin to reduce the efficacy of the other agents in the regimen, as well as a concern for creating clinical dysbiosis by administering too many antibiotics at once. All animals had three rounds of negative culture and parasitology results prior to declaration of GPF status and commencement of post-treatment sampling.

After establishing an effective treatment to eliminate EPs, we then implemented enhanced exclusion practices in an attempt to maintain a long-term GPF colony (see Materials and Methods). This was done based on a previous study demonstrating that macaque fecal microbiome gradually reverts towards baseline following antibiotic and fecal microbial transplantation [11], and clinical experience with the spread of EPs within and between rooms in animal facilities. We therefore wanted to evaluate our methodology to prevent a relapse of EPs and their potential confounding immunological effects. To assess efficacy of our EP exclusion practices, five sets of longitudinal fecal samples were collected, and microbiological and parasitic examinations were performed. Diagnostic results revealed study animals successfully maintained in a GPF status for > 6 months, indicating that the practices were successful in preventing exposure despite the presence of these agents in adjacent rooms in the facility (rhesus macaques in the adjacent room had the following EP prevalence: 23/27 (85%) *C*. *coli*, 5/27 (19%) *C*. *jejuni*, and 7/27 (26%) *S*. *flexneri*; no parasitology was performed on that cohort so the prevalence of parasites is unknown).

**Fig 1:**
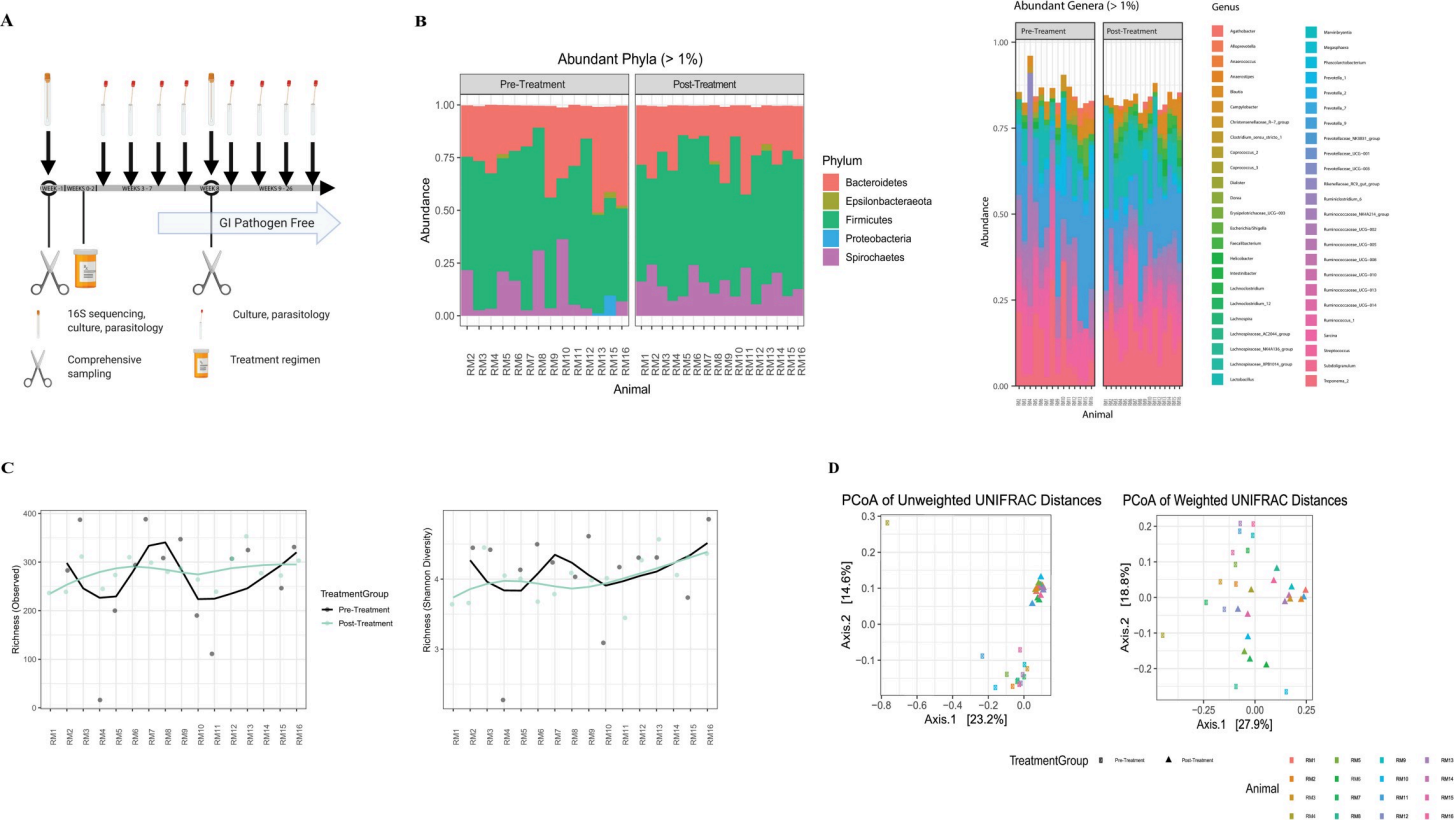
The multimodal therapy regimen does not cause microbiota diversity loss instead treatment decreases microbiota variation between individual study subjects. **(A)** Study design showing experimental timepoints for multimodal treatment administration, tissue and fecal sampling. Microbiota, fecal, parasitic assays and comprehensive surgical sampling performed (-1 week and 8 weeks), multi-modal therapy regimen (0–2 weeks), serial fecal and parasitic assays collected for remainder of study (2–26 weeks) (GPF after 3 consecutive negative results). **(B)** Gastrointestinal microbiota taxa distribution of the relative abundance of major (>1%) phyla (left) and (>1%) genera (right) comparing pre- and post-multimodal therapeutic regimen timepoints for study animals. Individual animals are represented by bar graphs and bacterial communities by a specific color designation (see figure key). **(C)** Comparison of alpha diversity by the measurement of observed richness (left) (p = .457) and Shannon diversity (right) (p = 0.875) of pre and post treatment timepoints for all study animals, statistical significance determined by paired two-way t-test. **(D)** Beta diversity measured by Principal Coordinate Analysis plots of the unweighted and weighted UniFrac of the pre- and post-treatment timepoints for all study animals, assessed by PERMANOVA (p = .001)(individual animals designated by color, study time points by shape (pre-treatment = o, post-treatment = Δ).

The fecal microbiome was compared pre- and post-treatment, by sequence analysis of DNA coding for the V4 hypervariable region of 16S ribosomal RNA, to determine if the short course multimodal therapeutic regimen resulted in less individual variation of phylogenetic taxa between animals. The predominant phylogenetic taxa were Firmicutes, Bacteroidetes, Spirochaetes, and Proteobacteria in all RM study cohorts (Fig 1B). Further t-test analysis of the relative abundance of Proteobacteria and family *Enterobacteriaceae* (bacterial populations associated with anti-microbial resistance) showed no evidence of enrichment with the studies’ multi-modal treatment regimen, but rather a decrease of the bacterial populations, although statistically insignificant (p = 0.144, p = 0.356). At the phylum and genus level, it is visually appreciable that the taxa distribution shows less individual variation following treatment (Fig 1B). Alpha diversity (measurement of species richness and/or diversity within a sample) did not significantly differ between pre- and post-treatment groups by quantitative and qualitative chao1 and Shannon diversity indices using a two-way paired t-test analysis (Fig 1C), although there is a considerable flattening of the post-treatment alpha diversity matrices curves, indicating that the treatment induces comparable diversity between the study subjects. Significantly increased beta diversity distance (measurement of differences between samples) and clustering of the pre- and post-treatment cohorts by the principal coordinate analysis of unweighted and weighted UniFrac metrices (permANOVA p = 0.001) (Fig 1D), further suggest that treatment created a more similar microbiome between study subjects, thereby reducing individuality.

**Fig 2:**
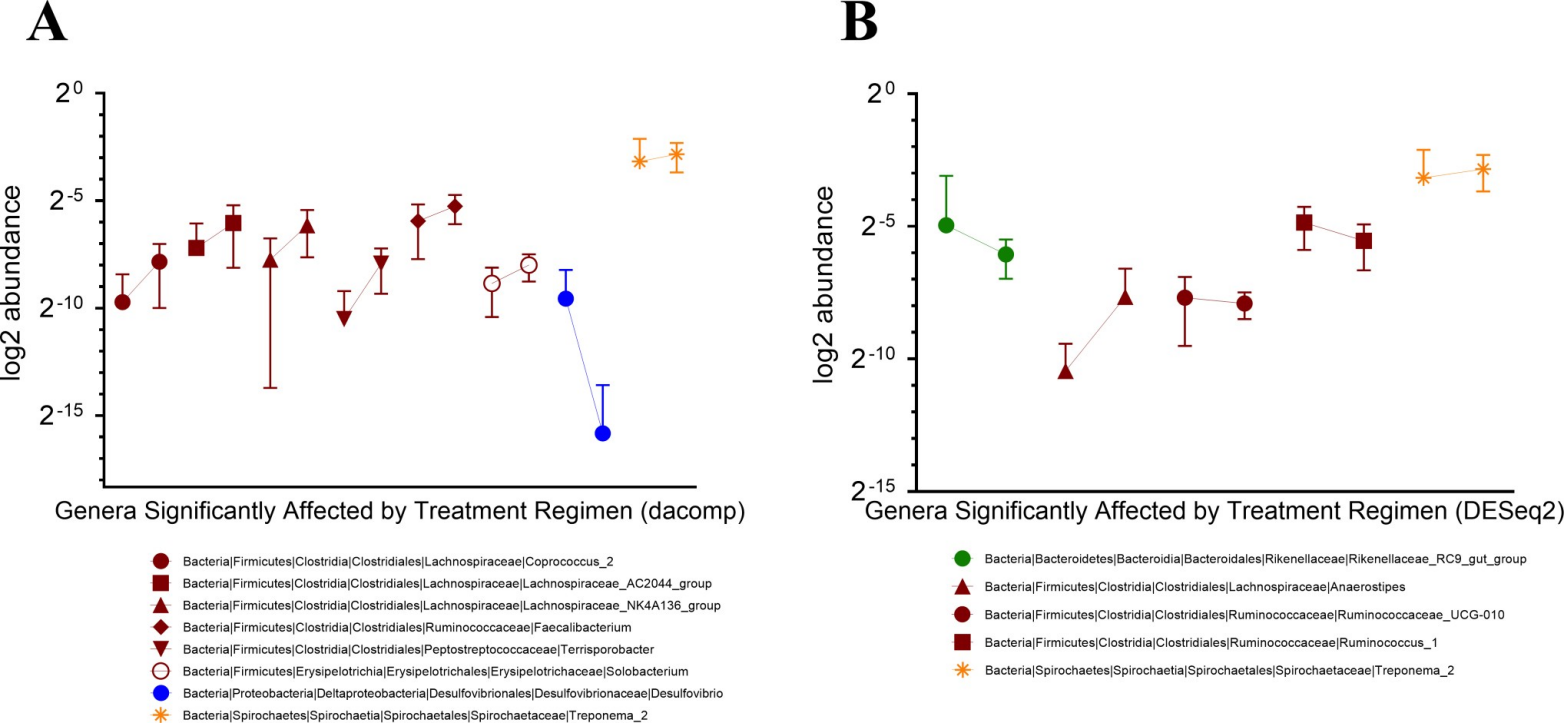
Multimodal therapy causes only a small subset of taxonomic genera to have microbiota community perturbances; enrichment of anti-microbial resistant Proteobacteria genera are not detected. **(A)** Dacomp and **(B)** DESeq2 analyses are used to assess for genera relative abundance differences for pre- and post-treatment timepoints for all study animals. Color distinguishes between different taxonomic phyla, shape/color distinguishes individual genera (see figure key), pre- and post-treatment timepoints are connected by a line for each genus. Genera classes jointly identified by the analyses are designated by (*). All genera differences represented by Dacomp and DESeq2 analyses are statistically significant (p-value <0.004).

**Fig 3:**
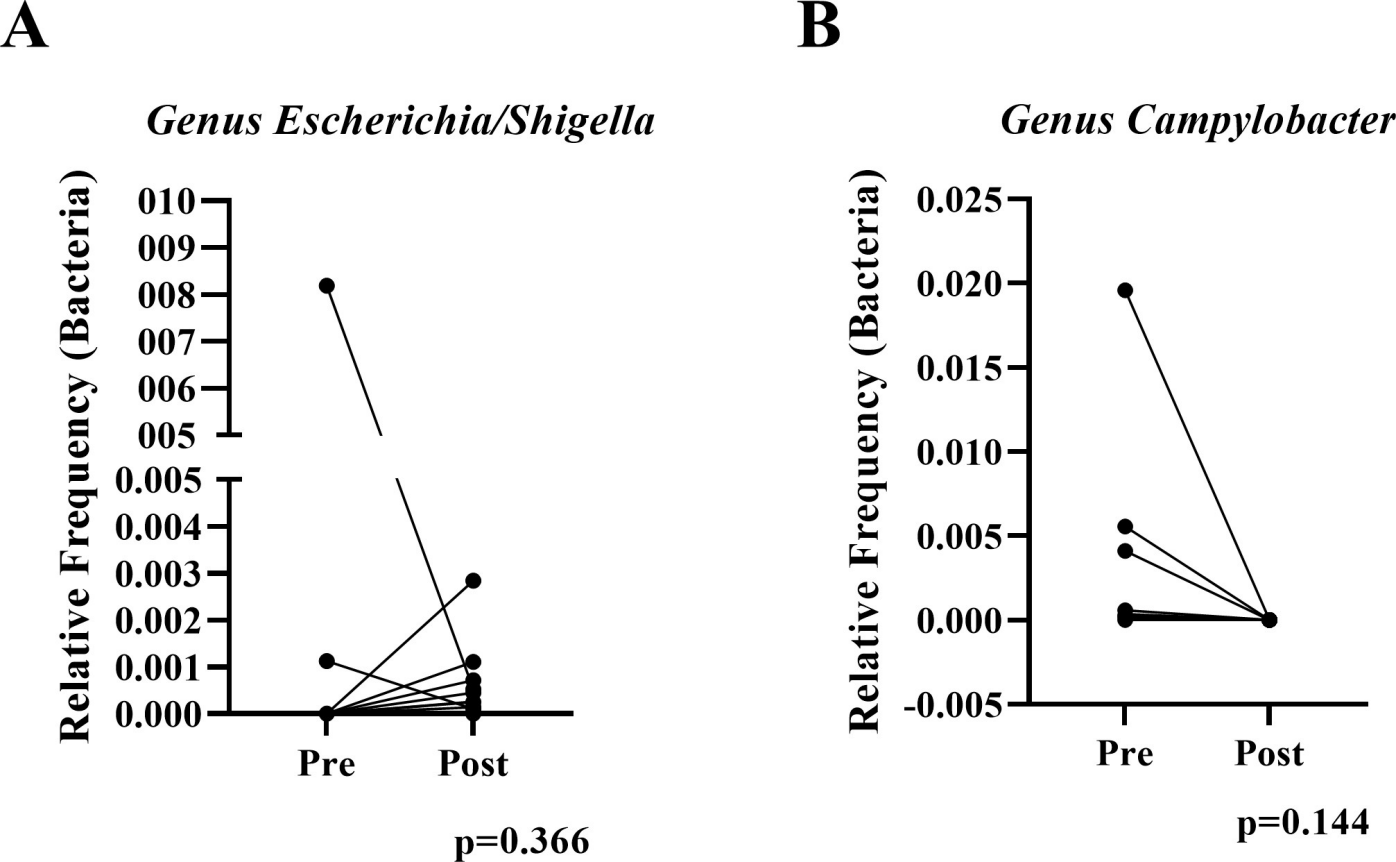
Multimodal therapy showed some bacterial community perturbances of *Escherichia/Shigella* and *Campylobacter*, although statistically insignificant, animals did remain culture free of pathogenic species post-treatment. **(A)** The relative abundance for genus *Escherichia/Shigella* comparing pre- and post-treatment regimen for study animals, assessed by paired t-test analysis. **(B)** The relative abundance of genus *Campylobacter* comparing pre- and post-treatment regimen for study animals, assessed by paired t-test analysis.

Following treatment, the most common taxonomic shifts involved Clostridia genera, and significant relative abundance perturbance of genus *Treponema*_2 were dually identified by DESeq 2 and dacomp taxonomic analyses (Fig 2). Specifically, when examining the relative abundance of the two specific bacterial pathogens that the treatment regimen aimed to reduce (*Campylobacter* spp., and *Shigella* spp.), we observed a reduction of the genus *Campylobacter* below the limit of detection by 16S sequencing, as well as the absence of *Campylobacter* and *Shigella* spp. by rectal culture. While our microbiome sequencing approach was unable to differentiate between *Shigella* from *Escherichia* (Fig 3), the absence of *Shigella* by rectal culture suggests that the remaining identified sequences likely belong to *Escherichia*. Collectively, these data indicate that our short course multimodal therapy successfully suppressed inter-animal phylogenetic differences, creating an increased similarity in fecal microbiota across study subjects.

### Short course multimodal treatment regimen improves gastrointestinal integrity and reduces gastrointestinal inflammation

**Fig 4:**
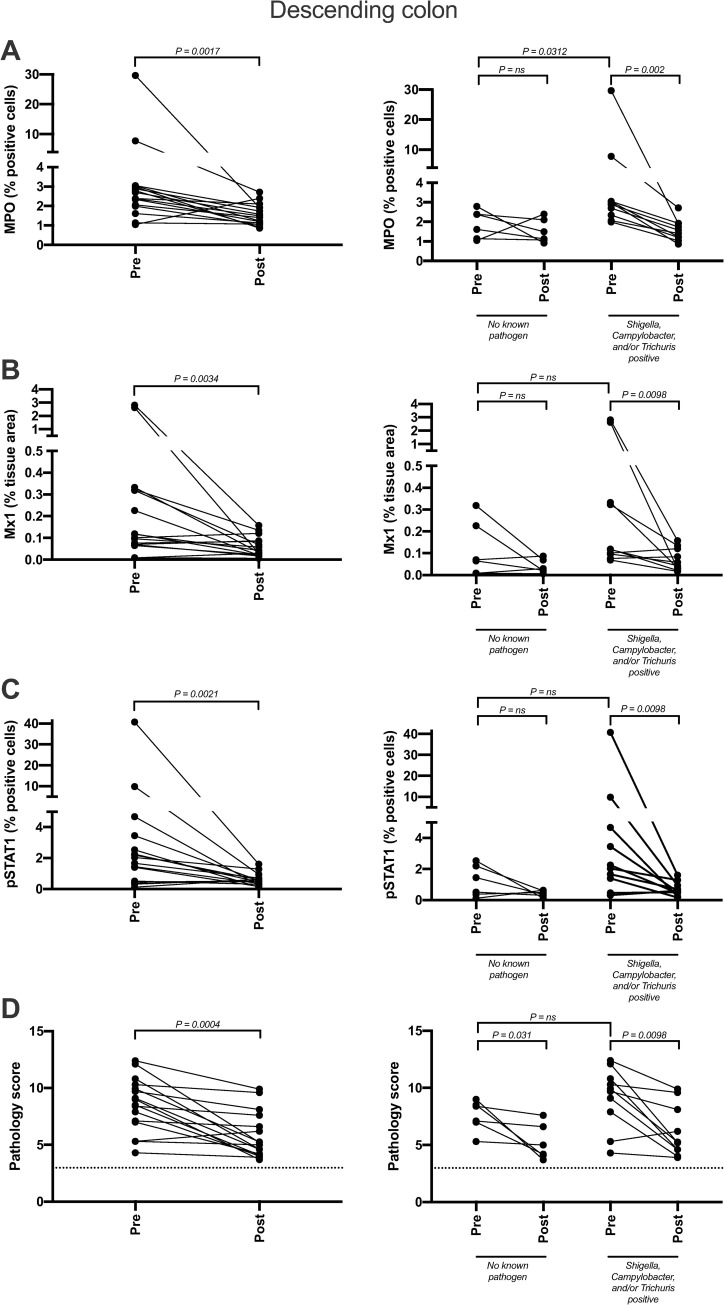
Inflammation and tissue pathology are reduced in the descending colon following treatment. Inflammation of formaldehyde-fixed, paraffin-embedded tissues was assessed via immunohistochemistry (IHC) and H&E. After the clean-up regimen, descending colon biopsies showed substantial reduction in several inflammatory pathways, including neutrophil recruitment (MPO; A), interferon signaling (Mx-1; B), cytokine signaling (pSTAT1; C), and overall pathology (D). IHC quantification was performed using HALO and pathology quantification occurred via independent scoring by two pathologists. The dotted line in panel D marks the minimum pathology score available, which is 3.

To evaluate distal descending colonic mucosal inflammation in study animals we utilized three IHC biomarkers (myeloperoxidase (MPO), phosphorylated signal transducer and activator of transcription 1 (pSTAT1), and type 1 IFN responsive gene product myxovirus resistance protein 1 (Mx1) as well as histologic scoring. Pre-treatment baseline mucosal samples of animals without identified EPs (n = 6), had significantly lower expression of MPO (p = 0.031) (Figs 4A and S1), a marker of neutrophil infiltration that directly correlates with reduced barrier integrity [4,12], in the colon lamina propria compared to animals with pre-existing EPs (n = 10). Importantly, following treatment and confirmation of GPF status, there were significant reductions in colonic neutrophil infiltration and mucosal inflammation (MPO p = 0.0017, Mx1 p = .0034, and pSTAT1 p = 0.0021) (Fig 4A, 4B, and 4C), which, combined with the reduction in histopathology score (p = 0.0004) (Fig 4D) indicating reduced inflammatory cell infiltration and improved mucosal and lamina propria architecture (S2 Fig), suggests elimination of EPs and/or the enrichment of beneficial taxa result in restoration of the GI-tract epithelial barrier and attenuates inflammation. Additionally, the inter-animal variability, as assessed by variance from the mean, was significantly reduced in both groups post-treatment (MPO p = 0.04, Mx1 p = .0038, and pSTAT1 p = 0.026). CD4+ T lymphocyte and CD68/CD163+ tissue macrophage populations remained unaltered, although the inter-animal variability of CD4+ T cells was significantly reduced after treatment (p = 0.0187) (S1 Fig).

### Short course multimodal treatment regimen results in decreased immune cell activation and proliferation in the GI-tract

We utilized 9-color and 14-color multiparameter flow cytometric immune surveillance panels to assess changes in innate and adaptive immune populations pre- and post-treatment. Changes in innate immune cell frequencies (specifically monocytes, pDC, and mDC) indicate host responses to foreign antigens and inflammation within tissues [13]. Furthermore, increased expression of CD169 on monocytes can indicate immune activation in response to pathogens, especially in the context of lentiviruses and adenoviruses. Following innate responses to pathogens, adaptive immune responses can be characterized by activation (increased CD38 expression)[14] and proliferation (increased Ki67 expression)[15] in T cell memory subsets.

**Fig 5:**
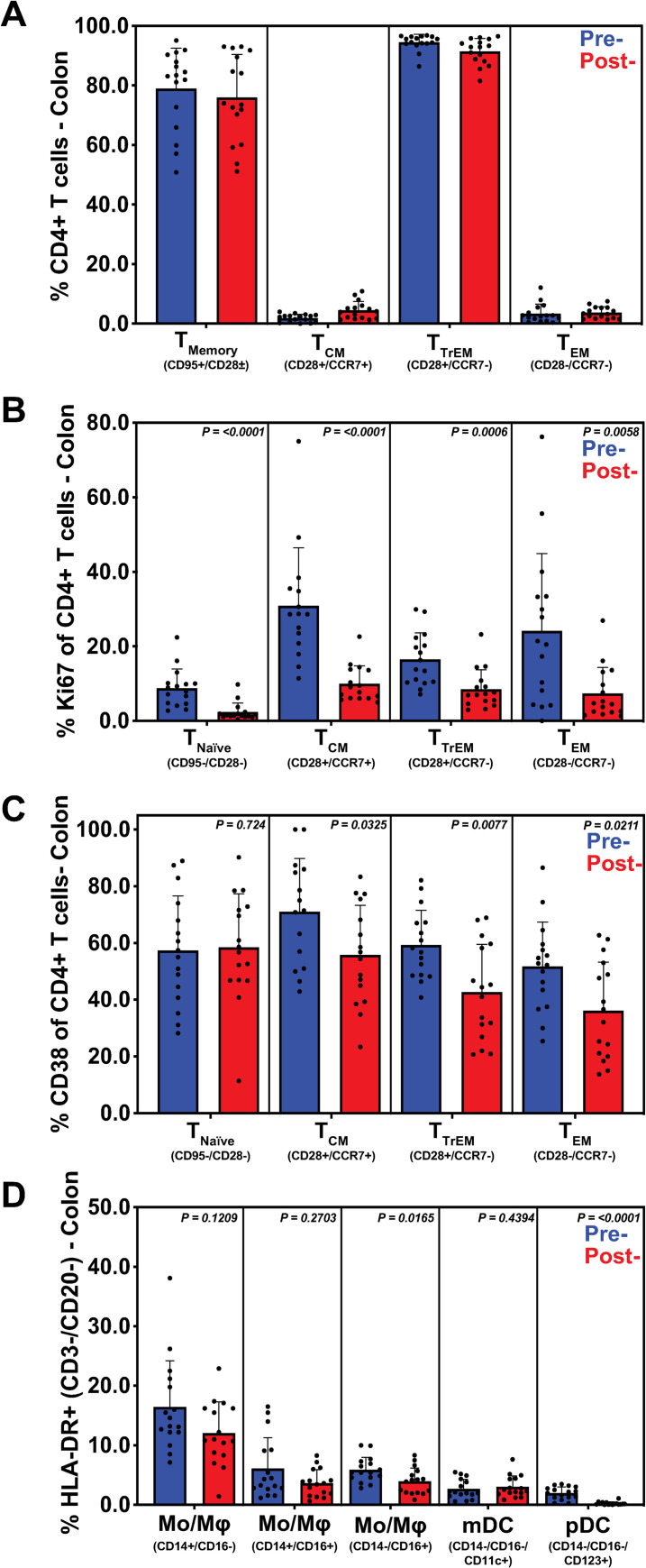
Multimodal treatment regimen restores decreased immune cell activation and proliferation in descending colon tissue. Flow cytometric analysis was performed on CD4+ T cells, dendritic cells (DC), and monocytes (MO) derived from descending colon biopsies, before and after multimodal treatment. Subsets were summed for overall responses with the figure showing the mean (+ SEM) of these overall responses both pre- and post-treatment. While CD4+ T cells did not exhibit any significant change in phenotype (**A**), there was a significant reduction in proliferation (Ki67) (**B**), as well as a reduction in activation (CD38) (**C**) in all T cell subsets. Additionally, innate immune cells showed significant reduction of CD14- CD16+ and pDC subsets, indicating reduced activation and DC gut trafficking, respectively (**D**). SEM reduction in measured T cell and innate subsets indicate a more normalized expression post-treatment. CD4+ T cell memory subsets were defined by expression of CD28, CD95 and CCR7. Monocytes and DCs were defined by forward and side scatter properties followed by selecting CD3-, CD20-, CD8-, HLA-DR+ populations. Monocytes were further distinguished by expression of CD14 and CD16 whereas DCs were defined by a lack of the two markers. DC subsets—myeloid and plasmacytoid—were further differentiated by their expressions of CD11c and CD123, respectively.

Following treatment and confirmation of GPF status, there were no significant changes in CD4+ T cell memory populations in the distal descending colon (Fig 5A). However, a significant reduction in proliferation (Ki-67+) of naïve (p<0.0001), T_CM_ (p<0.0001), T_TrEM_ (p = 0.0006), and T_EM_ (p = 0.0058) CD4+ T cell subsets was exhibited (Fig 5B). Additionally, the inter-animal variability in Ki-67+ CD4+ T cells was significantly reduced post-treatment (p = 0.0038). Finally, establishment of GPF status resulted in a decrease in CD38+ T_CM_ (p = 0.0325), T_TrEM_ (p = 0.0077) and T_EM_ (p = 0.0211) CD4+ T cells (Fig 5C). Reductions of non-classical CD14^low^CD16+ monocytes (p = 0.0165) and pDCs (p<0.0001) were also observed within the descending colon (Fig 5D). In agreement with previous reports [2], our results demonstrate background EPs cause local immune activation and inflammation with reduced mucosal integrity and barrier breaches. Regardless of initial EP status on rectal culture/parasitology, these data suggest that our short course multimodal therapy resulted in a more consistent mucosal immunological environment across study subjects, improved barrier integrity, and reduced local inflammation.

### Inflammation and immune activation at GI-tract draining sites are reduced following short course multimodal therapy

**Fig 6:**
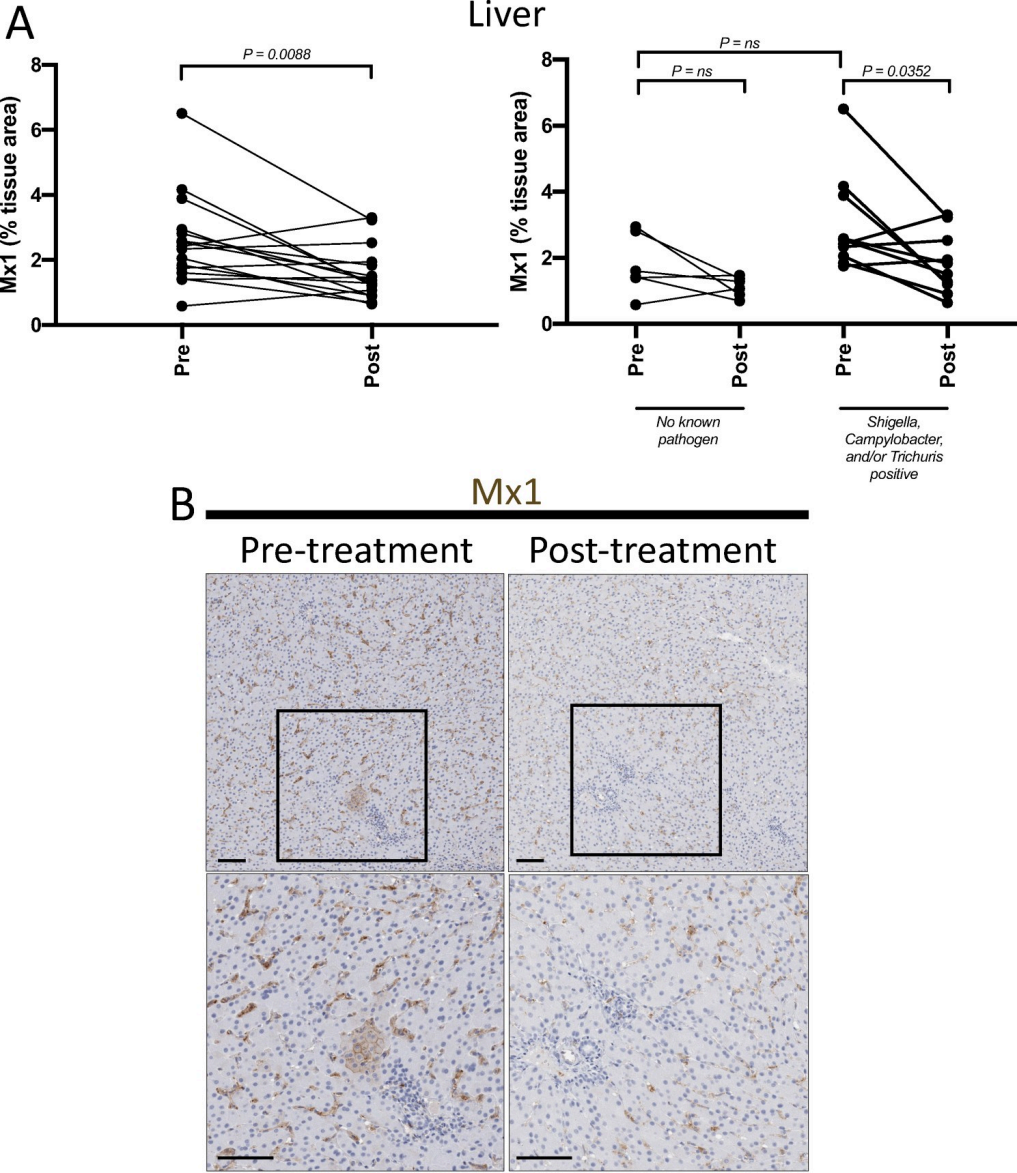
Reduction in inflammatory signaling pathways in the liver following multimodal therapy. Formaldehyde-fixed, paraffin-embedded tissues were assessed for Mx1, an interferon-induced GTPase, via immunohistochemistry (IHC). **(A)** Animals with known enteric pathogens had a larger range of liver Mx1 expression before treatment and significant reduction following the short course multimodal treatment. **(B)** Representative images of Mx1-stained pre-treatment sections of liver (left-hand column) exhibited higher focal staining within tissue parenchyma, as well as increased staining within cells residing in the sinusoids. Treatment (right-hand column) led to a substantial reduction in Mx1 within both of these tissue compartments (i.e. tissue parenchyma and sinusoid).

**Fig 7:**
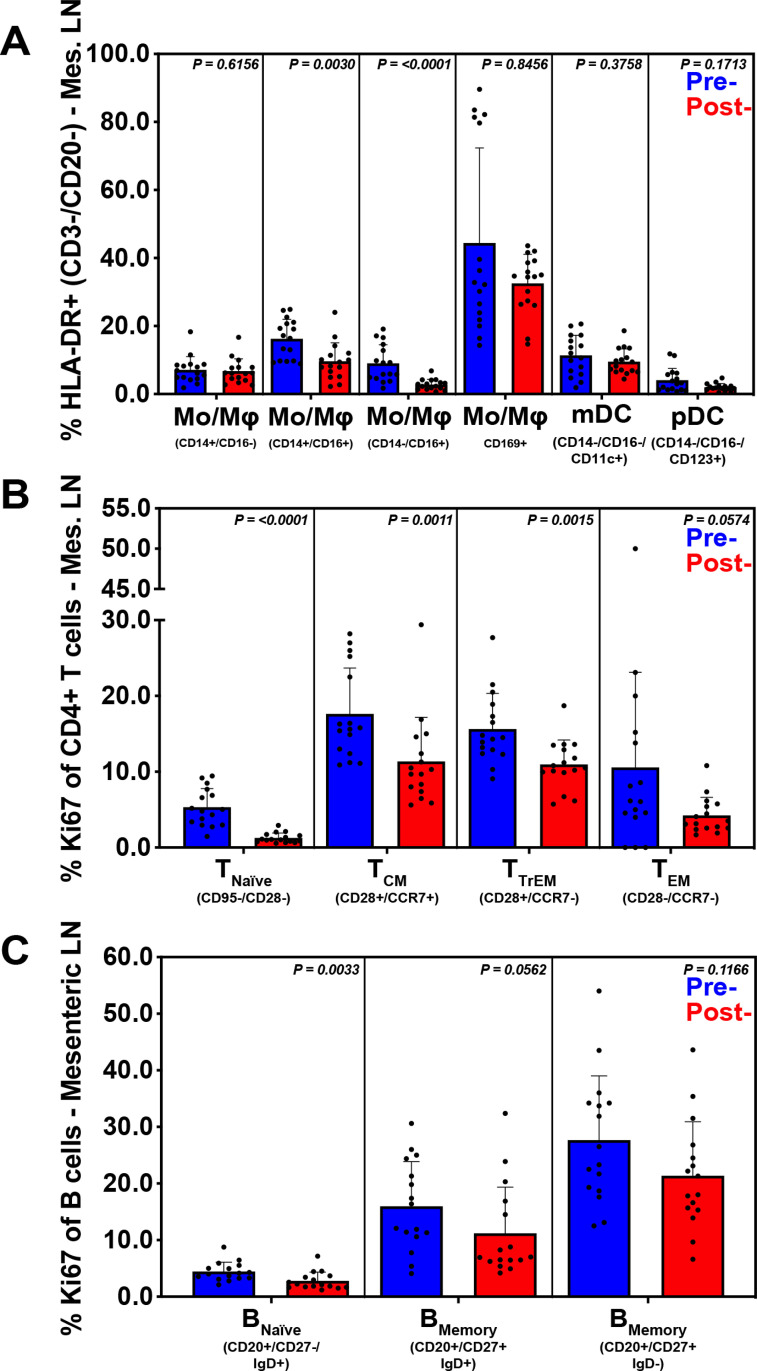
Inflammation and immune activation at GI-tract draining sites are reduced following short course multimodal therapy. Flow cytometric analysis was performed on B cells, CD4+ T cells, DCs, and MOs derived from mesenteric lymphoid tissue, before and after multimodal treatment. Subsets were summed for overall responses with the figure showing the mean (+ SEM) of these overall responses both pre- and post-treatment. A reduction in both CD14+ CD16+ and CD14- CD16+ monocytes was observed, as well as a decreased SEM for CD169+ expression by all HLA-DR+ cells (**A**). A reduction of the proliferation marker Ki67 and reduced SEM post-treatment in CD4+ T cells (**B**) and B cells (**C**) was exhibited. B cells were defined by their CD20 positivity followed by their expressions of CD27 and IgD. T cell and innate subsets were defined as described in Fig 5.

Mesenteric lymph nodes are the most proximal site to receive lymph draining from the GI-tract, while the liver is the first site to encounter GI-tract venous blood via the portal vein. These sites are potentially exposed to higher concentrations of leukocytes, inflammatory mediators, microbes and microbial products from the GI-tract when barrier breaches and inflammation are present. To evaluate the impact of treatment leading to GPF status on inflammation and immune activation we performed quantitative IHC (MPO and Mx1) and flow cytometry as described above with the addition of B cell phenotypes (memory, activation, and proliferation) which were possible due to the larger yield of lymphocytes from the MLN. Additionally, changes in CD21 expression [16] and IgM/IgG levels [17] were assessed and can be indicative of B cell maturation or isotype switching in response to ongoing antigen stimuli. Following treatment, there was a significant reduction in liver Mx1 expression (p = .0088) (Fig 6), but no significant changes within MLN. However, there was a significant reduction of intermediate CD14+CD16+ (p = 0.0030) and non-classical CD14-CD16+ (p<0.0001) monocytes from MLN following treatment (Fig 7A). Treatment leading to GPF status also resulted in a significant reduction of activated (Ki-67+), naïve (p<0.0001), T_CM_ (p = 0.0011), T_TrEM_ (p = 0.0015), and a trend toward reduction of activated T_EM_ (p = 0.0574) CD4+ T lymphocytes in MLN (Fig 7B), as well as a decrease in CD38+ T_CM_ (p = 0.0007) and T_TrEM_ (p = 0.0008) CD4+ T cells in MLN. Furthermore, treatment leading to GPF status also resulted in a significant reduction of Ki-67+ naïve B cell proliferation (p = 0.0033) and a trend for reduced Ki-67 expression among IgD+ and IgD- memory B cells (Fig 7C).

**Fig 8:**
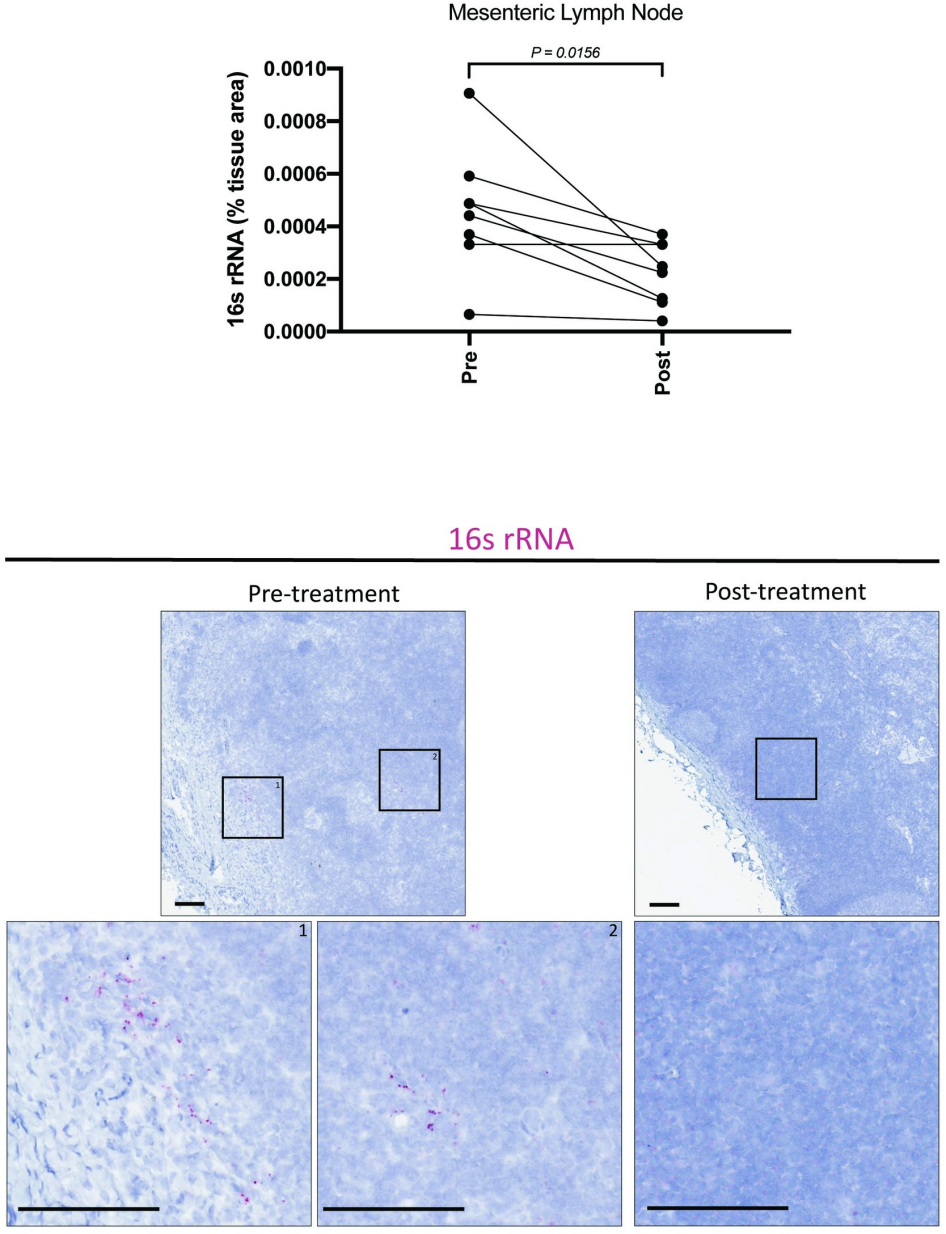
Microbial translocation to draining lymph nodes is reduced following short course multimodal therapy. RNAscope was performed on a subset of animals in which colonic or peripheral inflammation was observed via other markers (e.g. MPO, Mx1, pSTAT1, etc.), to quantify translocated bacteria within the inferior MLN using pan-reactive 16s rRNA probes. After treatment to induce GPF status, a marked decrease in 16s rRNA was observed in MLN, consistent with restoration of the GI epithelial barrier.

While neutrophil infiltration (MPO staining) in the GI lamina propria is a strong surrogate marker for local epithelial damage, we sought to directly quantify translocated microbiota in the draining lymphatic chain by assessing pan-bacterial 16s rRNA in the MLN (Fig 8), specifically focusing on the subset of animals displaying GI tract neutrophil infiltration or inflammation in any biopsy specimen prior to treatment leading to GPF status. Importantly, the number of translocated bacteria (as measured by the percent area of the MLN occupied by bacterial 16s rRNA) was significantly (p = 0.0156) reduced in animals following treatment to generate GPF status (Fig 8). In combination with our gastrointestinal data, reduction in hepatic Mx1, activated macrophages, and activation/proliferation of CD4+ T and B cells as well as reduced microbial translocation to MLNs following treatment leading to GPF status suggests this strategy leads to decreased exposure to pro-inflammatory signals (leukocytes, inflammatory mediators, microbes and microbial antigens) at these GI-tract draining sites due to elimination of EPs, resulting in improved integrity of the mucosal barrier.

### Elimination of EPs following short course multimodal therapy normalizes systemic inflammation and immune activation

**Fig 9:**
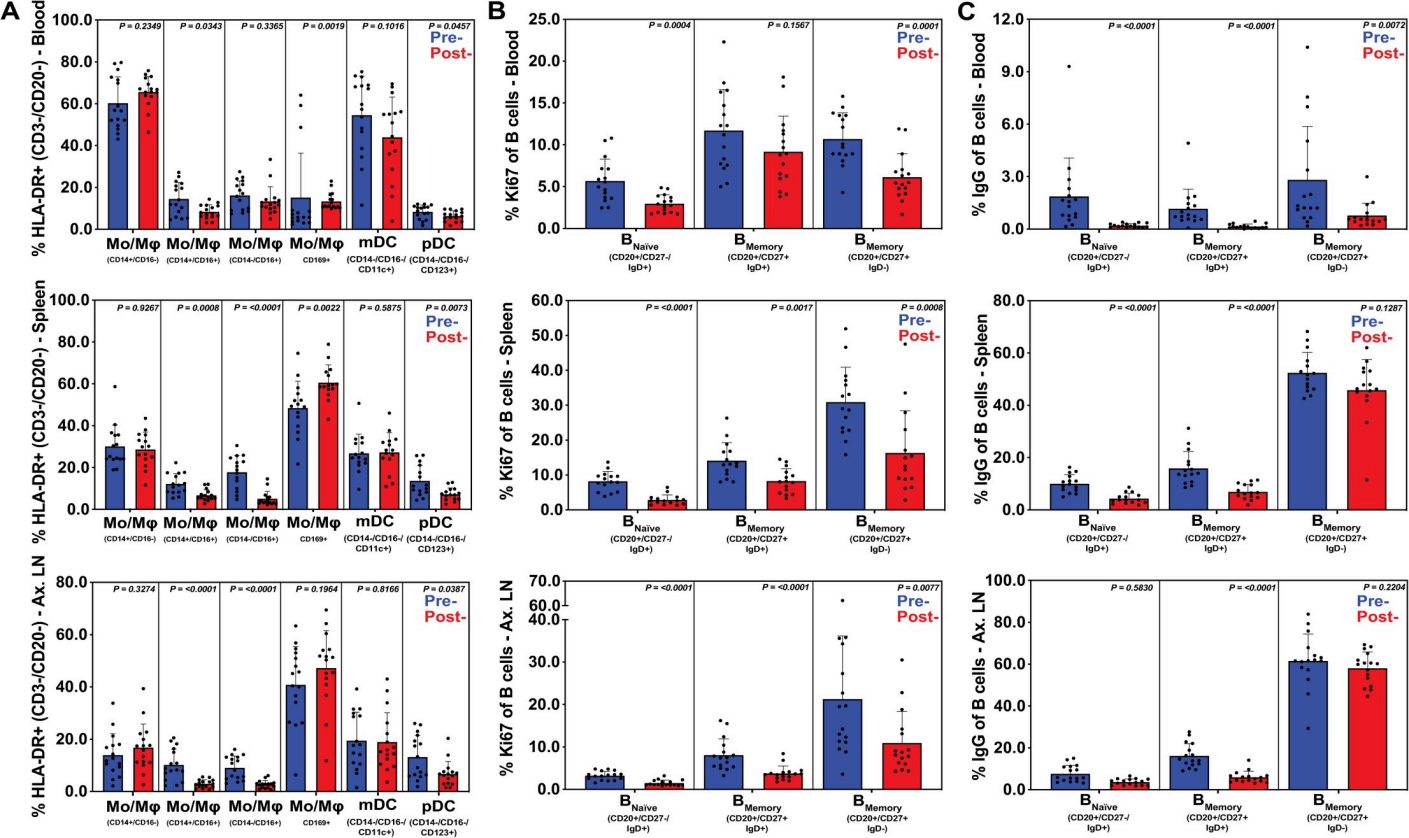
Elimination of EPs following short course multimodal therapy normalized systemic inflammation and immune activation in innate myeloid and B cells. Flow cytometric analysis was performed on B cells, DCs, and MOs derived from whole blood, spleen and axillary lymphoid (ALN), before and after multimodal treatment. Subsets were summed for overall responses with the figure showing the mean (+ SEM) of these overall responses both pre- and post-treatment. Reduced SEM was observed in most tissues with significant decreases in total CD16+ populations in spleen and ALN (**A**) along with significant reductions of pDCs in those tissues. Increased splenic CD169+ on myeloid cells was also observed (**A, panel 4**). A reduction in B cell expressions of the proliferation marker Ki67 (**B**) and IgG (**C**) in all memory subsets was observed in whole blood, spleen and ALN, showing reduced immune activity in those tissues after therapeutic treatment. Immune cell subsets were defined as described in Figs 5 and 7.

Finally, we evaluated systemic inflammation and adaptive immune activation pre- and post-treatment in peripheral blood, spleen, BAL, BM, and ALN samples using flow cytometric panels described above. Treatment leading to development of GPF status resulted in reductions of monocyte and pDC cell populations (Fig 9A), specifically, significantly lower proportions of intermediate CD14+CD16+ monocytes in peripheral blood (p = 0.0343), spleen (p = 0.0008) and ALN (p<0.0001), and a reduction of non-classical CD14-CD16+ monocytes in spleen (p<0.0001) and ALN (p<0.0001). As expected, peripheral blood also contained lower proportions of circulating CD169+ monocytes (p = 0.0019) as well as an influx of splenic CD169+ monocytes (p = 0.0022). Furthermore, post-treatment GPF status resulted in lower levels of pDCs in peripheral blood (p = 0.0457), ALN (p = 0.0387) and spleen (p = 0.0073), likely due to reduced antigen exposure and trafficking to these sites. No significant differences between pre- and post-treatment were observed in BAL or bone marrow.

After treatment leading to GPF status, a reduction of naïve B cell proliferation (Ki-67 expression) was evident in peripheral blood (p = 0.0004), ALN (p<0.0001) and spleen (p<0.0001) (Fig 9B). Reduced levels of Ki-67 expression were also present in IgD+ memory B cells in ALN (p<0.0001) and spleen (p = 0.0017), as well as on IgD- memory B cells in peripheral blood (p = 0.0001), ALN (p = 0.0077) and spleen (p = 0.0008) tissues. Furthermore, all B cell IgG responses (naïve p<0.0001; IgD- memory p = 0.0072; IgD+ memory p<0.0001) decreased in peripheral blood, and a similar trend occurred in IgD+ memory B cells in ALN (p<0.0001) and spleen (p<0.0001) tissues, as well as in naïve splenic B cells (p<0.0001) after treatment (Fig 9C).

**Fig 10:**
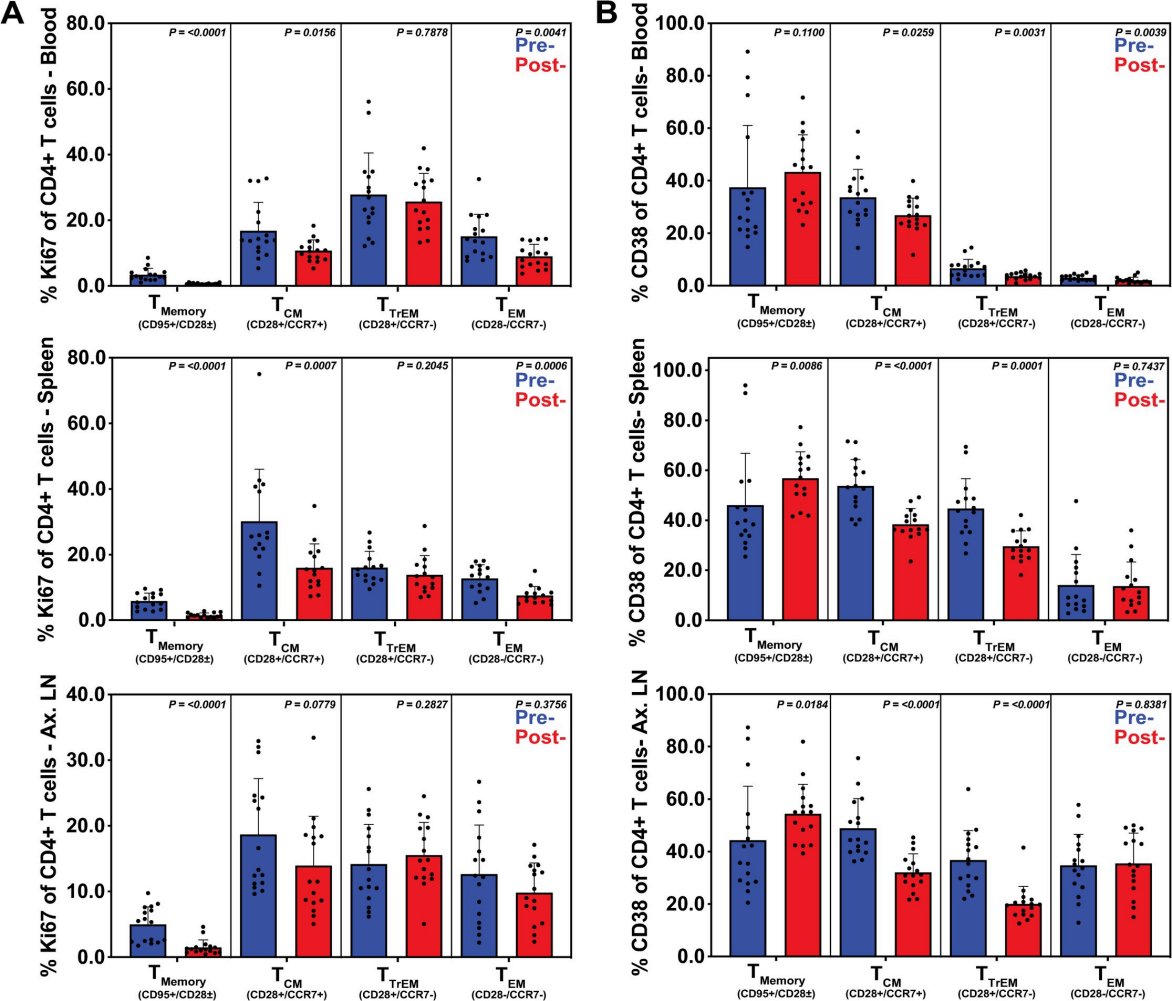
Elimination of EPs following short course multimodal therapy normalized systemic inflammation and immune activation in CD4+ T cells. Flow cytometric analysis was performed on CD4+ T cells derived from whole blood, spleen and axillary lymphoid (ALN) tissues, before and after multimodal treatment. Subsets were summed for overall responses with the figure showing the mean (+ SEM) of these overall responses for both pre- and post-treatment. Reduction in proliferation (Ki67) was observed in all tissues (**A**), especially in central and transitional memory subsets, as well as in whole blood and splenic effector memory populations. Decreased activation (CD38) was also observed in the central and transitional memory subsets in all three tissues (**B**) as well as in the effector memory subset in whole blood. Immune cell subsets were defined as described in Fig 5.

Evaluation of adaptive immune cell proliferation (Ki-67 expression) revealed a significant post-treatment reduction in the proportion of activated Ki-67+ CD4+ T lymphocytes in peripheral blood (naïve p<0.0001, T_CM_ p = 0.0156, T_EM_ p = 0.0041), and spleen (naïve p<0.0001, T_CM_ p = 0.0007, T_EM_ p = 0.0006), as well as decreased expression of Ki-67+ naïve CD4+ T lymphocytes in ALN (p<0.0001) (Fig 10A). We also observed changes in T cell activation with a decrease in CD38+ T_CM_ CD4+ T cells in blood (p = 0.0259), ALN (p<0.0001) and spleen (p = 0.0001); in T_TrEM_ CD4+ T cells in blood (p = 0.0031), ALN (p<0.0001) and spleen (p<0.0001) and in T_EM_ CD4+ T cells in blood (p = 0.0039) (Fig 10B).

Altogether, our data indicate reduced activation of local, draining, and systemic monocytes, dendritic cells, CD4+ T cells and B cells following treatment, which, combined with the histology, MPO and pan-bacterial 16s rRNA staining, indicates that these changes were due to decreased microbial antigen presentation as a result of improved integrity of the mucosal barrier.

## Discussion

We have demonstrated that creation and maintenance of gastrointestinal pathogen free (GPF) rhesus macaques for >6 months is possible using a short course multimodal therapy followed by enhanced animal care practices resulting in significantly reduced levels and inter-animal variability of inflammation at local, draining, and systemic sites, as well as improved colonic mucosal barrier function. With the goal of obtaining more stable baseline microbiome data, animals chosen for this interventional study had not received antibiotics for at least 2 months nor had evidence of diarrhea for at least 6 months prior to collection of baseline samples. As the vast majority of animals screened did not meet these selection criteria, the animals presented in this work represent a cohort of animals that is healthier than average at the ONPRC and likely minimized the potential to demonstrate a positive impact of the therapy. Despite this, we demonstrated that GPF status resulted in improved mucosal barrier function as evidenced by histopathology and reduced levels of MPO+ neutrophils in the distal descending colon by IHC, as well as reduced pan-bacterial 16s rRNA in GI tract draining MLNs. We also demonstrated reduced intermediate (CD14+CD16+) and non-classical monocytes (CD14-CD16+), reduced populations of peripheral dendritic cells, Ki-67+ and CD38+ CD4+ T cells, Ki-67+IgG+, and Ki-67+IgD+ B cells. These findings indicate lower levels of background inflammation in the distal descending colon, draining mesenteric lymph node, and systemically in peripheral blood, spleen, and axillary lymph node.

We also demonstrated reduced inflammation in the distal descending colon (Mx1, pSTAT1) and liver (Mx1) via IHC. The inter-animal variability of inflammatory and immune activation markers was reduced resulting in a more consistent baseline between research animals upon which to evaluate the impact of experimental manipulations. More consistent outcomes in NHP studies could possibly reduce the number of animals required to achieve statistical significance in models of immunology, inflammation, and infectious diseases. The importance of these changes for SIV macaque models was demonstrated by a proof of concept study where the IR SIVmac239X AID50 was 100-fold higher in animals that had received a multimodal treatment regimen similar to what was used to generate the GPF animals. Unfortunately, we did not assess EPs or levels of inflammation or disruptions in the mucosal barrier in these proof of concept animals. Other animals from the same shipment/source as the proof-of-concept animals had positive results for *Shigella* spp., *Strongyloides* spp., *Balantidium* spp. and *Entamoeba* spp., prior to treatment and post-treatment, for the cohort that received the short course multimodal therapy, the proof of concept animals were housed in a facility which excluded *Shigella* spp., and parasitic EPs (*Entamoeba* spp., *Balantidium* spp., *Strongyloides* spp., and *Trichuris* spp.), and were longitudinally confirmed free of EPs through serial diagnostic sampling. However, in our follow-up study the changes were largely consistent regardless of detected background EP status prior to treatment. One limitation of the follow up study is that we performed a single set of assessments (rectal culture, fecal parasitology, and microbiome assessment) prior to initiation of the short course multimodal therapy. We deemed it likely that any EPs that were significantly influencing the local, draining and systemic inflammatory state would be present at high enough levels to be detected on a single exam. In a majority of study animals, the impact of pre-study cleanup was more significant in animals with demonstrable EPs. However, the benefit of cleanup when comparing inter-animal variability was clearly present, irrespective of the background EP status. Thus, it is strongly suggestive there were either additional EPs not detected by the single pre-treatment assessment or that additional bacterial/parasitic EPs that are not recognized by our standard assessments were present. For example, *Escherichia coli* spp. which have been reported to have ~25% prevalence in some macaque colonies, can cause opportunistic GI disease [2], and require specialized assays to detect the presence of virulence genes that differentiate them from normal flora [18], would not have been detected but could have been eliminated or reduced in prevalence by our treatment.

Microbiota play an influential role on host health, with beneficial bacterial communities (i.e. probiotics (*Lactobacillus* spp.)) having gastro-protectant properties [19] and enrichment of pathobiont bacterial populations correlating with local (e.g., Crohn’s disease) and systemic (e.g., Alzheimer’s, rheumatoid arthritis) diseases [20–22]. One of the aims of this study was to determine if using a short course multimodal therapeutic regimen (paromomycin, fenbendazole, azithromycin, and enrofloxacin) would control for differences between the gastrointestinal microbiota of study subjects. In contrast to previous literature that utilized a variety of antibiotics (paromomycin, clindamycin, cephalexin, vancomycin +/-/or enrofloxacin) in immune-competent or compromised macaques [23–25], our study’s therapeutic regimen did not result in a loss of microbiota diversity, which is the most common indicator of intestinal dysbiosis [26], however our study did not include untreated temporal controls for comparison. Manuzak et al demonstrated that antibiotic (enrofloxacin, paromomycin, clindamycin, or cephalexin) related microbiota loss was transient and recovered by day 28 post-administration [25]. Therefore, our data may not have captured this transient loss due to sampling at 6 weeks post-treatment. Similar to previous reports, our beta diversity pCoA results demonstrate that treatment increased study subject clustering and a definitive difference was shown between individual baseline and post-treatment time points, representing a decrease in study subject inter-individual variability and demonstrating microbial community perturbation between study time points [23–25]. The macaques in this study showed no phylogenetic phyla differences with treatment, with the predominant phyla being Firmicutes, Bacteroidetes, Spirochaetes, and Proteobacteria across study subjects. Phylogenetic bacterial community shifts were present at the genus level. Dacomp and DEseq2 analyses conjointly identify perturbances of species, *Treponema_2*, and the order *Clostridiales* (gram negative obligate anaerobes) as the most common genus population showing disturbances. Therefore, this study’s short course multimodal therapeutic regimen reduced inter-individuality between microbiota of study subjects with only modest levels of genera perturbation. With the use of a multimodal antibiotic and parasitic treatment, we did not see higher proportions of *Enterobacteriaceae* which is linked to multi-drug resistance and can have pro-inflammatory properties [23]. Consistent with our results, previous literature have found paromomycin and azithromycin did not result in Proteobacteria enrichment rather a phylum reduction [25,27] likely explaining why expansion of Proteobacteria was not seen with our multimodal treatment. Importantly, through longitudinal diagnostic testing and expanded procedure practices we were able to achieve long-term exclusion of EPs, establishing a GPF colony. To our knowledge, this is the first demonstration that rhesus macaques can be maintained free of *Campylobacter* spp. for an extended period of time. The microbiota overall shows less individuality and appears to control for overt bacterial community outliers within study subjects. This, in combination with the beneficial effects of controlling for underlying immune activation and inflammation, as well as the added benefit of occupational protection from common zoonotic EPs, makes for an ideal macaque for a number of research models. Antibiotic resistance could become an issue, however several factors help to mitigate this risk; 1) to date we have not detected any of the pathogenic bacteria we are attempting to eliminate after completion of the therapeutic regimen, 2) though an in-depth assessment has not been performed, we have not observed increased rates of antibiotic resistance in other bacterial populations in treated animals when clinical samples are taken for procedures such as wound care, though the number of samples to date in treated cohorts is quite small, and 3) as these models are terminal, and given the practices employed, there is little risk of transmitting resistant bacteria to other animals/cohorts if they were to occur. Further studies are needed to evaluate how quickly the microbiome stabilizes after the regimen ends.

Rhesus macaques are commonly used as models for human immunodeficiency virus (HIV) and there are established SPF4 and SPF9 breeding colonies to meet the need for Indian origin rhesus macaques for these studies. Studies have demonstrated that for HIV as well as pathogenic SIV infections, the GI-tract is the major site for pathogenesis despite the route of acquisition. During the SIV/HIV acute phase, the gut mucosa and lamina propria undergo immunological and cellular disruptions, increased mucosal permeability, damage, and microbial translocation, which perpetuates chronic immune activation and disease progression [12,28–30]. Pathogenesis can be impacted by background levels of colitis as evidenced in elite controller macaques and African Green Monkeys (AGM) where administration of DSS resulted in increased viral loads and increased viral loads with loss of mucosal CD4+ T cells, respectively [4].

Disruptions of mucosal barriers and mucosal inflammation have also been shown to promote HIV/SIV acquisition. A prior macaque study [31] showed that levels of Ki-67+ effector memory CD4+ T cells correlated with increased acquisition and higher peak viremia in an intrarectal SIV challenge model. Importantly, in this study we demonstrate that treatment resulted in significantly reduced Ki-67+ effector memory CD4+ T cells, as well as reduced inter-animal variability in GPF animals. It is well documented that bacterial, parasitic and viral sexually transmitted infections (STIs) promote HIV acquisition in humans when present at the site of mucosal exposure (oral, vaginal, and/or rectal) due to disruptions in barrier integrity and increases in activated CD4+ T cells [32]. Other ulcerative colitis-inducing agents have additionally been shown to correlate with HIV status [33]. It has been shown for instance that men who have sex with men with HIV are at greater risk for *Entamoeba histolytica* infection, but the data might also support the conclusion that barrier breaches and inflammation associated with *E*. *histolytica*, which were well-documented by colonoscopy, augmented activated CD4+ T cell populations and increased the risk of rectal HIV acquisition [34]. In fact, it was recently shown that an experimental animal’s colony of origin had a significant impact on a SHIV macaque model in which animals from two different source colonies, presumably with different prevalence of EPs, had a per challenge acquisition rate of 47% versus 21% when identically challenged [35].

In conclusion, we have demonstrated the ability to create and maintain macaques free of common bacterial and parasitic GI pathogens using the gold standard assays (rectal culture and fecal flotation/wet mount parasite exams) for these agents. These GPF macaques have improved GI mucosal barrier function, which leads to a reduction in local, draining, and systemic inflammation and reduced inter-animal variability across a host of parameters. This reduced variability could produce more consistent outcomes both between cohorts within an institution as well as between institutions, leading to improved rigor and reproducibility in key preclinical macaque models. There is also the potential to achieve statistically significant results using fewer animals which can both serve to reduce animals utilized as called for in the 3R’s [36] and reduce costs in these models. Finally, as these agents can lead to clinical disease that can be severe and debilitating, preventing them also serves as a refinement per the 3Rs, meets the expectations of the Guide for the Care and use of Laboratory animals (ref preventive medicine section) [37], and augments animal welfare.

## Materials and methods

### Ethics statement

All nonhuman primates were maintained at AALAC-accredited facilities, and animal research was performed in accordance to institutional policies and approved by the Institutional Animal Care and Use Committee.

Study animals, treatment regimen description, and timeline of tissue collection procedures.

### Proof of concept study assessing the impact of the multimodal treatment regimen on acquisition of SIVmac239X

A total of 18 healthy male Indian-origin rhesus macaques between 3 and 6 years of age were used in accordance to the policies of the Institutional Animal Care and Use Committee at the National Cancer Institute (NCI), an AAALAC-accredited institution, which abides by the USDA Animal Welfare Regulations [38] and the Guide for the Care and Use of Laboratory Animals [37]. Animals were socially-housed during the study in accordance with the IACUC protocol and facility practices. All enrolled macaques were research naïve, had not received antibiotics for at least 2 months, and were SPF (serologically negative for simian T-lymphotropic virus-1, SIV, simian type D retrovirus, and *Macacine* herpes 1) prior to the start of the study. Animals were genotyped for common MHC Class I alleles using allele-specific PCR as previously described [39] Mamu-A*01, -B*08 and -B*17 animals were excluded from this study. Throughout the study, all animals were uniformly fed Purina LabDiet 5045 (Purina Mills International, St. Louis, MO), daily nutritional enrichment items (grains, fruits, or vegetables), ad libitum access to water, and were not exposed to additional pre-, pro-, or anti-biotic therapeutics after receiving the study-related treatment regimen. Animals assigned to the treatment regimen were administered courses of enrofloxacin (10 mg/kg intramuscularly (IM) every 24 hours for 10 days), paromomycin (25 mg/kg orally (PO) every 12 hours for 10 days), and fenbendazole (50 mg/kg PO every 24 hours for 5 days) administered simultaneously as was done in the follow up study, and a cage change occurred on day 8 or 9 to ensure no pathogens would be reintroduced from the cage. No further exclusion practices were required in this facility as all animals were treated with this regimen prior to being admitted to the facility, and testing showed the facility to be free of *Shigella* spp., and parasitic EPs. Using transfection derived SIVmac239X [40], 18 macaques were challenged intrarectally (IR) with varying doses of SIVmac239X in 3 x 1 ml volume of RPMI. Animals without pre-treatment (n = 4) were challenged with either 1x10^3^ or 3x10^3^ infectious units (IU) as determined by TZM-bl assay (reference no. 8129; NIH AIDS Research and Reference Reagent Program). Animals treated with the multimodal therapy (n = 16) were challenged intrarectally with either 3x10^3^, 3x10^4^, or 3x10^5^ IU in the same 3 x 1 ml volume. IR SIVmac239X challenge was performed as previously described [41]. Blood draws were obtained twice weekly post-challenge and infection status determined both by a positive quantitative real-time PCR outcome as previously described [42] and by single genome sequencing of plasma as described below.

### Assessment of the multimodal treatment regimen

An additional 16 healthy male Indian-origin rhesus macaques between 3 and 6 years of age were used in accordance to the policies of the Institutional Animal Care and Use Committee at the Oregon National Primate Research Center (ONPRC), an AAALAC-accredited institution, which abides by the USDA Animal Welfare Regulations [38] and the Guide for the Care and Use of Laboratory Animals [37]. All animals were captive-born at ONPRC and inhabited socially-housed indoor/outdoor enclosures before their transfer to indoor standardized housing prior to study start and socially-housed during the study in accordance with the IACUC protocol and facility practices. All enrolled macaques were research naïve, had not received antibiotics for at least 2 months, had no GI disease requiring antibiotics for at least 6 months, and were SPF (serologically negative for simian T-lymphotropic virus-1, SIV, simian type D retrovirus, and *Macacine* herpes 1) prior to the start of the study. Animals were genotyped for common MHC Class I alleles using allele-specific PCR as previously described [24,39]. Mamu-A*01, -B*08 and -B*17 animals were excluded from this study. Throughout the study, all animals were uniformly fed Purina LabDiet 5000 (Purina Mills International, St. Louis, MO), daily nutritional enrichment items (grains, fruits, or vegetables), ad libitum access to water, and were not exposed to additional pre-, pro-, or anti-biotic therapeutics after receiving the study-related treatment regimen. Prior to treatment all animals were assessed for background EP status by rectal culture (intrarectal sample (BBL CultureSwab)), fecal parasitology (free catch collection), and by fecal microbiota analysis (via sterile rectal loop, placed directly into a collection conical and placed on dry ice until stored at -80 degrees Celsius prior to processing). Animals assigned to the treatment regimen were administered courses of enrofloxacin (10 mg/kg IM every 24 hours for 10 days), paromomycin (25 mg/kg PO every 12 hours for 10 days), fenbendazole (50 mg/kg PO every 24 hours for 5 days), and azithromycin (40 mg/kg PO for first day followed by 20 mg/kg PO once a day for 4 days). The enrofloxacin and paromomycin were started together on day 1, fenbendazole was added on day 6, and a cage change occurred on day 8 or 9 to ensure no pathogens would be reintroduced from the cage. The azithromycin began the day after completion of the other 3 drugs (i.e. day 11). Three animals received an additional round of fenbendazole 6 weeks prior to the post-treatment sampling due to a *Trichuris* spp. positive parasitology result in two of the animals, and concern for exposure of a socially housed-partner. Consistent with the definition of SPF and eSPF where animals are only considered SPF/eSPF after they are removed from any exposure to non-SPF animals and have at least two rounds of negative tests post last potential exposure [1], animals were only considered GPF once they had been treated with the multimodal treatment regimen, tested to ensure negative status, and were no longer exposed to any non-GPF animals post treatment. Again, as with SPF/eSPF the level of certainty of GPF status increases with additional time and testing post last potential exposure. All animals had three rounds of negative culture and parasitology results prior to declaration of GPF status and post-treatment sampling. All enrolled study animals had baseline samples of spleen, mesenteric lymph nodes (MLN), and liver by a minimally invasive laparoscopic surgery [43], scopeless bronchoalveolar lavage (BAL) [44], microbiome fecal collection, axillary lymph node (ALN), bone marrow, and distal descending colonic mucosal pinch biopsies taken prior to administration of the treatment. These samplings were repeated 6 weeks post-administration of the last dose of the additional round of fenbendazole. Additionally, animals were followed by culture and parasitology for 6 months following the last round of fenbendazole to ensure maintenance of GPF status.

### Description of practices to maintain gastrointestinal pathogen free (GPF) status

We established GPF animal biosecurity practices (based in large part on our successful eSPF practices) to exclude common EPs in macaque research colonies. GPF animal rooms were positioned in a location to limit work traffic and designated as first entry rooms to promote optimal compliance for facility workflow and further prevent EP fomite contamination. The area in front of the animal rooms was partitioned off by a taped line to create a physical barrier and serve as a multipurpose work area which functioned as GPF PPE, husbandry and research (i.e., food, equipment) storage, and staff/animal workspace. Upon entering the demarcated GPF workspace, all staff don new/clean GPF-designated PPE, including new Tyvek suits, boot covers, gloves, face shields, masks, and hair bonnets. All consumables used in the study were new or clean prior to animal distribution and stored separately from the rest of the animal colony. All non-consumable enrichment, research, husbandry, and diagnostic equipment were new or disinfected prior to animal introduction or contact. Cages and husbandry equipment were transferred directly from the clean cage wash to the animal rooms with casters disinfected as they crossed into the tape-designated clean area. Surgical equipment and procedural rooms were disinfected prior to study-related use and GPF animals underwent planned procedures prior to other colony animals. Once animals were assigned to a GPF study, their electronic record alerted veterinary staff to consult the principal investigator prior to prescribing pre-, pro-, or anti-biotic medications to prevent study-confounding variables. To reduce physiological stress, animals were acclimated to designated GPF rooms 4 weeks prior to study start.

### Flow cytometry assays

Peripheral blood, bone marrow, BAL, spleen, ALN, MLN, liver and descending colon pinch biopsy samples were obtained as described above and were processed then stained for flow cytometric analysis. Peripheral blood was collected in an ACD BD Vacutainer (BD, 364606). ALN, MLN, spleen, BAL, and bone marrow were collected in R10 media (RPMI-1640 with 10% sterile filtered Newborn Calf Serum, 1% penicillin-streptomycin, 1% L-glutamine, 1% sodium pyruvate, and 50 nM/mL of 2-Mercaptoethanol) at biopsy. ALN, MLN and spleen were processed using a pestle and screen for CD-1 (Sigma-Aldrich, S1020-5EA) in a tissue culture dish. Processed tissues were strained through a 40 μm filter into a 50 mL conical, centrifuged at 700 rcf for 10 minutes, and resuspended in R10 before obtaining the cell yield using a Horiba ABX Pentra 60 C+ hematology analyzer. Bone marrow was processed similarly to the above tissue, with omitting the CD-1 screen.

Liver and descending colon biopsy tissues were processed using a gentleMACS Octo Dissociator with Heaters (Miltenyi Biotech). The tissue samples were incubated in 12 mL of R3 media (same as R10 but with 3% sterile filtered Newborn Calf Serum) supplemented with 300μg/mL DNase-I (Roche Applied Science, REF# 10104159001) and 150 μg/mL collagenase (Sigma, C9722), aliquoted into a gentleMACS C tube (Miltenyi Biotech, Auburn, CA). The dissociator was programmed to agitate two sets of clockwise/counter-clockwise at 300 rpm for 20 sec and at 50 rpm for 40 min following by another round of clockwise/counter-clockwise 20 sec agitation at 37°C. The isolated cells were strained through a 70 μm filter, centrifuged at 700 rcf for 10 minutes, and resuspended in R10 media before obtaining the cell yield using a Horiba ABX Pentra 60 C+ hematology analyzer.

Flow cytometric assays were performed on an LSR II Becton Dickinson instrument. Data were analyzed using the FlowJo software program (version 9.9.6; Ashland, OR: Becton, Dickinson and Company; 2020). Each assay used 1x10^6^ lymphocytes per biopsy tissue and/or 100 μL of peripheral blood.

T cells were defined by CD3 positivity and small lymphocyte light scatter. CD3+ T cells were further defined by CD4 and CD8 positivity. Memory and naïve T cell subsets were delineated based on CD28, CCR7, and CD95 expression patterns on gated CD4^+^ or CD8^+^ T cells. Each subset was defined as: Naïve: CD28+ CD95- CCR7-, Central Memory (T_CM_): CD28+ CD95+ CCR7+, Transitional Memory (T_TrEM_): CD28+ CD95+ CCR7-, and Effector Memory (T_EM_): CD28- CD95+ CCR7-. B cells were defined by CD20 positivity and small lymphocyte light scatter. Memory and naïve B cell subsets were defined by CD27 positivity (memory) with additional subsets defined by IgD expression (IgD+ and IgD-). Monocyte subsets were defined by expression of CD14 and CD16 (classical: CD14+ CD16-, intermediate: CD14+ CD16+ and non-classical: CD14- CD16+), while myeloid (mDC) and plasmacytoid (pDC) dendritic cells were defined by the lack of CD14 and differing expressions of CD11c and CD123, respectively.

### Antibodies

The following fluorophore-conjugated antibodies were used in this study: from BD Biosciences: anti-CD4 (L200; BUV395), anti-CD8a (SK-1; BUV737 and BV510), anti-CD28 (CD28.2; BV510), streptavidin (BV605), anti-CD21 (B-ly4; BV711), anti-IgG (G18-145; BV786), anti-Ki67 (B56; FITC), anti-IgM (G20-127; PE-CF594), anti-CD123 (7G3; PerCP Cy5.5) and anti-CD3 (SP34-2; Alexa Fluor 700); from BioLegend: anti-CCR7 (G043H7; Biotin), anti-CD27 (M-T271; BV421), anti-CD16 (3G8; Pacific Blue), anti-CD14 (M5E2; FITC), anti-CD169 (7–239; PE), anti-HLA-DR (L243; PE-Dazzle), anti-CD95 (DX2; PE-Cy7), anti-CD11c (3.9; APC), and anti-CD20 (2H7, APC/Fire 750); From Novus: anti-CD38 (AT1;APC); From SouthernBiotech: anti-IgD (polyclonal; PE).

### T cell gating hierarchy

Whole blood and tissue-derived lymphocytes were stained using fluorophore conjugated monoclonal antibodies and analysis was performed using a LSR II Becton Dickinson instrument. The resulting list mode multi-parameter data files were analyzed using the FlowJo software program (version 9.9.6; Ashland, OR: Becton, Dickinson and Company; 2020) with the hierarchy shown here. CD3^+^ small lymphocytes were selected and divided into CD4^+^ and CD8^+^ T cell subsets, then defined as memory and naïve T cell subset populations based on CD28 and CD95 expression patterns. Memory subsets were then further differentiated by their expressions of CD28 and CCR7. Naïve (CD28^+^ CD95^-^), central memory (CD28^+^ CD95^+^ CCR7^+^), transitional memory (CD28^+^ CD95^+^ CCR7^-^), and effector memory (CD28^-^ CD95^+^ CCR7^-^) T cells were then analyzed for subsets manifesting upregulation of the markers for activation (CD38) and proliferation (Ki67) (S3 Fig).

### B cell gating hierarchy

Whole blood and tissue-derived lymphocytes were stained and analyzed using the software described above. CD20^+^ small lymphocytes were selected and divided into memory (IgD^+/-^ CD27^+^) and naïve (IgD^+^ CD27^-^) B cell subsets. The subsets were further analyzed for upregulation of activation marker CD38, proliferation marker Ki67, complement receptor CD21, as well as surface expression of immunoglobulins G and M (S4 Fig).

### Dendritic cell, monocyte/macrophage hierarchy

Whole blood and tissue-derived lymphocytes were stained and analyzed using the software described above. CD3^-^ CD20^-^ HLA-DR^+^ lymphocytes were selected and divided into monocyte/macrophage populations based on CD14 and CD16 expression. Dendritic cells were further defined by their lack of CD14 and CD16 as well as their expression of CD11c (myeloid DC) and CD123 (plasmacytoid DC). Upregulation of the activation marker CD169 was also measured on all HLA-DR^+^ lymphocytes (S5 Fig).

### Immunohistochemistry and quantitative image analysis

Tissues were fixed in freshly prepared 4% paraformaldehyde for 24 hours, transferred to 70% ethanol, and paraffin embedded within 5 days. Slides (5 μm sections) were deparaffinized in xylene and rehydrated through a series of graded ethanol to distilled water. Heat induced epitope retrieval (HIER) was performed with the antigen retrieval buffers citrate (Thermo, pH 6.0, AP-9003-500; for MPO), citraconic anhydride (0.01% containing 0.05% Tween-20; for Mx1), or Tris-HCl (0.01M, pH 8.6; for pSTAT1) in a Biocare NxGen Decloaking Chamber at 110°C for 15 minutes, cooled for 20 minutes, then rinsed twice in ddH2O and 1x TBS with 0.05% Tween-20 (TBS-T). Slides were incubated with blocking buffer (TBS-T with 0.25% casein) for 30 minutes at room temperature, rinsed with TBS-T, and incubated at room temperature with antibodies against MPO (Dako; Cat. No. A0398), Mx1 (EMD Millipore Cat. No. MABF938; clone M143/CL143), or pSTAT1 (Cell Signaling; Cat. No. 9167L), which were diluted in blocking buffer. Endogenous peroxidases were blocked with 1.5% H2O2 in TBS-T for 10 minutes. Slides were then incubated with Rabbit Polink-1 HRP (GBI Labs; Cat. No. D13-110; for MPO), Mouse Polink-2 HRP (GBI Labs; Cat. No. D37-110; for Mx1), or Rabbit Polink-2 HRP (GBI Labs; Cat. No. D39-110; for pSTAT1). Slides were developed using Impact DAB (3,3′-diaminobenzidine; Vector Laboratories), washed in ddH2O, counterstained with hematoxylin, mounted in Permount (Fisher Scientific), and scanned at 20x magnification on an Aperio AT2 (Leica Biosystems).

### Histopathologic examination and scoring

Colonic pinch biopsies were fixed in 4% paraformaldehyde for 24 hours and transferred to 80% ethanol at 4°C for 24–72 hours until processed and embedded in paraffin. Paraffin sections were cut at 5 μm and serially dehydrated and de-paraffined before routine staining with hematoxylin and eosin. Tissue sections were blindly assessed by standard light microscopy by two pathologists using Leica DFC495 microscopes. There was an average of six biopsy per section, and each slide contained two sections. Five random, non-consecutive 40x fields selected to contain both mucosa and submucosa were evaluated (using the scoring schema below). Scores from the five fields were averaged, and any additional comments added.

**Table:** 

Inflammatory cell infiltrate	
Location	
1 = Mucosa only	
2 = Mucosa +/- segmental submucosa or +/- segmental crypt epithelium	
3 = Mucosa + moderate crypt epithelium + submucosa	
4 = Mucosa + crypt epithelium + crypt abscesses +/- submucosa	
Type	
1 = Lymphoplasmacytic +/- minimal eosinophilic	
2 = Lymphoplasmacytic + minimal histiocytic +/- mild to moderate eosinophilic	
3 = Lymphoplasmacytic + mild histiocytic +/- mild to moderate neutrophilic and eosinophilic	
4 = Predominantly neutrophilic and/or histiocytic + lymphoplasmacytic	
Severity (% infiltrate per 40x field)	
1 = Minimal (<10%)	
2 = Mild (10–25%, infiltrating, no expansion)	
3 = Moderate (25–50%, dense infiltrates +/- mild expansion)	
4 = Marked (>51%, dense infiltrates + expansion of lamina propria)	
Epithelial/lamina propria changes	
Hyperplasia	
0 = None	
1 = Minimal hyperplasia	
2 = Mild hyperplasia, mild goblet cell loss, +/- rare mitoses in upper 1/3rd of crypts	
3 = Moderate hyperplasia, moderate elongation of crypts, moderate mitoses in upper third of crypt epithelium +/- moderate goblet cell loss	
4 = Marked hyperplasia, many mitoses in upper third of crypts, +/- marked goblet cell loss, +/- crypt atypia	
Epithelial necrosis	Superficial extracellular/intrahistiocytic cellular debris
0 = none	0 = none
1 = Mild epithelial degeneration (apoptosis)	1 = Minimal
2 = Focal erosion +/- epithelial degeneration	2 = Mild
3 = Moderate erosion	3 = Moderate (diffusely superficial +/- mild expansion)
4 = Marked erosion +/- focal ulceration	4 = Marked (expansion of lamina propria, increased depth)

### 16S rRNA mesenteric lymph node RNAscope in situ hybridization and analysis

RNAscope in situ hybridization was performed as previously described using pan-reactive 16s rRNA probes (ACD Cat. No. 414461). In brief, after slides were deparaffinized in xylene and rehydrated through a series of graded ethanol to distilled water, retrieval was performed for 30 min in ACD P2 retrieval buffer (ACD Cat. No. 322000) at 95–98°C, followed by treatment with protease III (ACD Cat. No. 322337) diluted 1:10 in PBS for 20 min at 40°C. Slides were then incubated with 3% H_2_O_2_ in PBS for 10 min at room temperature. Prior to hybridization, probes stocks were centrifuged at 13,000 rpm using a microcentrifuge for 10 min, then diluted 1:2 in probe diluent (ACD Cat. No. 300041) to reduce probe aggregation tissue artifacts. Slides were developed using the RNAscope 2.5 HD Detection Reagents-RED (ACD Cat. No.322360), counterstained with CAT-hematoxylin (Biocare), mounted in Permount (Fisher Scientific), and scanned at 40x magnification on an Aperio AT2 (Leica Biosystems). Quantitative image analysis was performed using HALO software (v3.0.311.405; Indica Labs) on two inferior MLNs sections from each analyzed animal, pre-treatment and post-treatment. The ISH v.3.4.3 module was used to detect 16s rRNA and is presented as a proportion of total LN tissue (area/μm^2^). Manual curation was performed on each sample to ensure the annotations were accurate and to correct false positives.

### 16S isolation and analysis

16S rDNA was isolated from stool and sequenced on the MiSeq Illumina platform as previously described [45]. Raw Illumina FASTQ files were first demultiplexed using a custom Python script. Returned paired-end FASTQ reads were filtered and processed using the DADA2 package (v1.14.1) in RStudio (v1.1.463) to infer Amplicon Sequence Variants (ASVs) at a 99% identity threshold using the Silva database (v132). Before quality trimming 5,608,297 reads were included in 32 samples with an average of 175,259 reads per sample. Reads were trimmed to 200bp and filtered to exclude sequences with degenerate bases (N), more than 2 expected errors (maxEE), or chimerism. DADA2 quality trimming resulted in 2,333,726 reads for all the samples with an average of 72,929 reads per sample. Three samples with less than 1000 reads and 3 resultant unpaired samples were omitted from further analysis. ASVs identified as non-bacteria, mitochondria (Rickettsiales Mitochondria), and Cyanobacteria were removed from further consideration as were resultant genera at less than 3% prevalence or with no Family diversity. Fully analyzed 16S miSeq data (n = 26) are deposited in the NCBI Sequence Read Archive (SRA) under project number PRJNA633758.

### Nucleic acid extraction and quantification/single genome sequencing

Viral RNA from blood was sequenced using a combination real-time, SGA and Sanger sequencing described previously [40]. Briefly, a 300-bp portion of the integrase gene surrounding the mutated site was amplified and sequenced by a limiting-dilution PCR where only a single genome is amplified (SGA) per reaction [46]. vRNA was extracted with the QIAamp vRNA minikit (Qiagen) and then reverse transcribed into cDNA with SuperScript III reverse transcriptase according to manufacturer’s recommendations (Invitrogen). The cDNA was mixed with 1× reverse transcription (RT) buffer, 0.5 mM each deoxynucleoside triphosphate, 5 mM dithiothreitol, 2 U/ml RNaseOUT (RNase inhibitor), 10 U/ml Superscript III reverse transcriptase, and 0.25 mM antisense primer SIVmacIntR1 (5′-AAG CAA GGG AAA TAA GTG CTA TGC AGT AA-3′) and incubated at 50°C for 60 min and at 55°C for 60 min, heat inactivated at 70°C for 15 min, and then treated with 1 U of RNase H at 37°C for 20 min. PCR was then performed with a reaction mixture of 1× PCR buffer, 2 mM MgCl2, 0.2 mM each deoxynucleoside triphosphate, 0.2 μM each primer, and 0.025 U/μl Platinum Taq polymerase (Invitrogen) in a 10 -μl volume. Real-time PCR was performed with sense primer SIVmacIntF1 (5′-GAA GGG GAG GAA TAG GGG ATA TG-3′) and antisense primer SIVmacIntR3 (5′-CAC CTC TCT AGC CTC TCC GGT ATC C-3′) under the following conditions: 1 cycle of 94°C for 2 min and 40 cycles of 94°C for 15 sec, 55°C for 30 sec, 60°C for 1.5 min, and 72°C for 30 s. Template-positive reactions were determined by real-time PCR with SIVIntP 5′-TCC CTA CCT TTA AGA TGA CTG CTC CTT CCC CT-3′ with 6-carboxyfluorescein and ZEN/Iowa Black Hole Quencher (Integrated DNA Technologies) and directly sequenced with SIVmacIntR3 by Sanger sequencing using BigDye Terminator technology (Life Technologies).

### Statistical analyses

Sequencing abundance and diversity were assessed using the Phyloseq package (v1.30.0) in RStudio. Alpha diversity was quantified as Observed richness and Shannon diversity, with significance assessed by paired, two-way t-test. Beta-diversity was assessed by unweighted and weighted Unifrac, with statistical significance assessed by PERMANOVA. The empirical identification of differentially abundant bacterial taxa was determined using the DESeq2 (v1.26.0) and dacomp (v1.22) packages in RStudio. Reported abundance significance results from paired testing and incorporates package-specific p-value adjustments for multiple-comparisons. DESeq2 was run utilizing the likelihood ratio test function and dacomp using the Wilcoxon function. No data that met minimum threshold requirements as outlined in these Materials and Methods were excluded.

For statistical comparisons from flow cytometric data, unpaired t-tests followed by Mann-Whitney test was performed using GraphPad Prism version 8.0.0 (macOS, GraphPad Software, San Diego, California USA, www.graphpad.com). For statistical comparisons from IHC and histology data, either Wilcoxen matched-pairs signed rank tests (pre-treatment/post-treatment) or Mann-Whitney tests (intergroup comparisons) were performed again using GraphPad Prism version 8.0.0. Inter-animal variability was determined using the Lavene’s test performed using QI Macros Statistical Software for Excel (KnowWare International Inc. Denver, Colorado USA, qimacros.com).

## Supporting information

S1 Fig Inflammation is reduced, and T cell infiltration normalized, in the descending colon following treatment. **(A)** Representative images of formaldehyde-fixed, paraffin-embedded descending colon biopsies demonstrate the dramatically reduced neutrophil recruitment (MPO) observed following the clean-up regimen. **(B)** IHC quantification of CD4+ T cells and macrophages (CD68/CD163) was performed using HALO, which demonstrated markedly reduced inter-animal variance (P = 0.0187) assessed by Wilcoxon signed-rank test. **(C)** Despite a negligible change in overall IHC staining (CD4+ and CD68/CD168) between pre and post-treatment groups (scale bars = 100μm).
(PDF)

S2 Fig IHC and histological measures of colonic inflammation significantly reduced following treatment. Representative images of Mx1, pSTAT1, and H&E-stained sections of colon exhibiting the pre-and post-treatment changes observed with treatment designed to create GPF status. Pre-treatment sections (left-hand column) had higher staining for **(A)** Mx1, (B) pSTAT1, as well as **(C)** increased severity of inflammation and spread to the submucosa (**asterisk**), increased epithelial hyperplasia and necrosis **(arrowhead)**, and more superficial macrophages with intracytoplasmic cellular debris. Some high-scoring sections had crypt abscesses **(arrow)**. Post-treatment, minimal **(A)** Mx1 and **(B)** pSTAT1 were observed, and **(C)** low-scoring sections were relatively quiescent with normal submucosal gut-associated lymphoid tissue **(dotted line)** and low numbers of lamina propria lymphocytes.
(PDF)

S3 Fig T cell gated hierarchy. Whole blood and tissue-derived lymphocytes were stained using fluorophore conjugated monoclonal antibodies and analysis was performed using a LSR II Becton Dickinson instrument. The resulting list mode multi-parameter data files were analyzed using the FlowJo software program (version 9.9.6; Ashland, OR: Becton, Dickinson and Company; 2020) with the hierarchy shown here. CD3^+^ small lymphocytes were selected and divided into CD4^+^ and CD8^+^ T cell subsets, then defined as memory and naïve T cell subset populations based on CD28 and CD95 expression patterns. Memory subsets were then further differentiated by their expressions of CD28 and CCR7. Naïve (CD28^+^ CD95^-^), central memory (CD28^+^ CD95^+^ CCR7^+^), transitional memory (CD28^+^ CD95^+^ CCR7^-^), and effector memory (CD28^-^ CD95^+^ CCR7^-^) T cells were then analyzed for subsets manifesting upregulation of the markers for activation (CD38) and proliferation (Ki67).
(PDF)

S4 Fig B cell gated hierarchy. Whole blood and tissue-derived lymphocytes were stained and analyzed using the software described above. CD20^+^ small lymphocytes were selected and divided into memory (IgD^+/-^ CD27^+^) and naïve (IgD^+^ CD27^-^) B cell subsets. The subsets were further analyzed for upregulation of activation marker CD38, proliferation marker Ki67, complement receptor CD21, as well as surface expression of immunoglobulins G and M.
(PDF)

S5 Fig Dendritic cell, monocyte/macrophage hierarchy. Whole blood and tissue-derived lymphocytes were stained and analyzed using the software described above. CD3^-^ CD20^-^ HLA-DR^+^ lymphocytes were selected and divided into monocyte populations based on CD14 and CD16 expression. Dendritic cells were further defined by their lack of CD14 and CD16 as well as their expression of CD11c (myeloid DC) and CD123 (plasmacytoid DC). Upregulation of the marker CD169 was also measured on all HLA-DR^+^ lymphocytes.
(PDF)
