# Correction: Mitigation of endemic GI-tract pathogen-mediated inflammation through development of multimodal treatment regimen and its impact on SIV acquisition in rhesus macaques

**DOI:** 10.1371/journal.ppat.1011343

**Published:** 2023-04-19

**Authors:** Rachele M. Bochart, Kathleen Busman-Sahay, Stephen Bondoc, David W. Morrow, Alexandra M. Ortiz, Christine M. Fennessey, Miranda B. Fischer, Oriene Shiel, Tonya Swanson, Christine M. Shriver-Munsch, Hugh B. Crank, Kimberly M. Armantrout, Aaron M. Barber-Axthelm, Charlotte Langner, Cassandra R. Moats, Caralyn S. Labriola, Rhonda MacAllister, Michael K. Axthelm, Jason M. Brenchley, Brandon F. Keele, Jacob D. Estes, Scott G. Hansen, Jeremy V. Smedley

The published article [1] presents data from two separate animal studies. The first study conducted at the National Cancer Institute (NCI) comprised an 18-rhesus macaque (RM) intrarectal dose titration experiment to determine appropriate dosing of SIVmac239X for use in viral transmission/early necropsy studies. This notice addresses errors in the methodological description for the initial NCI study: in these animals, the authors did not assess gastrointestinal pathogens, microbiome, immunology, or mucosal barrier integrity, and the handling and treatment of the animals differed from that employed in the subsequent study conducted at Oregon National Primate Research Center (ONPRC). The originally published Materials and Methods section incorrectly reported some similarities between the animal handling and treatment regimens used in the two studies. This Correction provides amended methodological information, including that the multimodal therapeutic regimen for the NCI study included only metronidazole and fenbendazole, while the regimen for the ONPRC study included enrofloxacin, paromomycin, fenbendazole, and azithromycin.

## Initial dose titration study at NCI

The terms “proof of concept study” and “proof of concept animals” are inaccurate and are replaced in all instances with “initial dose titration study at NCI” and “initial NCI dose titration study animals”, respectively.

In the Materials and Methods section originally titled Proof of concept study assessing the impact of the multimodal treatment regimen on acquisition of SIVmac239X, the correct text is as follows:

Initial dose titration study at NCI assessing the impact of the multimodal treatment regimen on acquisition of SIVmac239X

A total of 18 male Indian-origin rhesus macaques between 2.6 and 13.4 years of age were used in accordance with the policies of the Animal Care and Use Committee at the NCI, an AAALAC-accredited institution, which abides by the USDA Animal Welfare Regulations [38] and the Guide for the Care and Use of Laboratory Animals [37]. Animals were singly housed in accordance with the IACUC-approved protocol and facility practices for the NIH facilities where the work was conducted (NIH main campus, Bethesda, MD or NIH Animal Center, Poolesville, MD). All enrolled macaques were from specific-pathogen free breeding colonies (Alpha Genesis/Morgan Island, NICHD, PrimGen, or Wisconsin National Primate Research Center) and were confirmed serologically negative for simian T-lymphotropic virus-1, SIV, simian type D retrovirus, and Macacine herpesvirus 1 prior to acquisition. All animals were negative for Mamu-B*08 and -B*17 alleles, while animals 4317 and 4402 were Mamu-A*01 positive. No animal received antibiotics in the 2 months prior to administration of the multimodal regimen. All animals were uniformly fed Purina LabDiet 5045 (Purina Mills International, St. Louis, MO) with daily nutritional enrichment items (grains, fruits, or vegetables), had ad libitum access to water, and received clinical care as needed. In order to conform with the USDA Animal Welfare Regulations to reduce the total number of animals used in this study, in some cases, animals were rechallenged after a prior lower dose challenge had clearly failed to result in infection. Initially, 4 RMs (ZD47, DD36, ZD49, ZD76) housed at the NIH Animal Center at Poolesville were challenged intrarectally with 3 ml of SIVmac239X in RPMI 1640 containing either 1x10^3^ (n = 2) or 3x10^3^ (n = 2) infectious units (IU) as determined by TZM-bl assay (reference no. 8129; NIH AIDS Research and Reference Reagent Program) as previously described [41]. Productive infection was documented in both animals at the higher dose and 1 of 2 at the lower dose based on quantitative RT-PCR from serial blood draws [42]. Additionally, the animals challenged with 3x10^3^ IU contained 6 and 8 distinct viral lineages based on genetic sequencing as previously described [40]. With 100% infection rate and multiple founder lineages per animal, plans were therefore made for early necropsy study using the same 3x10^3^ IU dose. Prior to challenge, the remaining 14 animals were transported to Bethesda, MD, and according to facility protocols, received treatment intended to reduce or eliminate common GI pathogens endemic in many RM populations. These animals were not confirmed GI pathogen free (GPF) based on the criterion of three rounds of post-treatment fecal culture and parasitology as employed for the subsequent ONPRC study for the assessment of the multimodal treatment regimen. The NCI study animals were categorized as “treated”, rather than “GPF”. Treatment for the NCI animals consisted of metronidazole 50 mg + 21 mg/kg orally twice daily for at least 10 days (which replaced the paromomycin normally used in this protocol due to lack of availability of paromomycin at the time of dosing for these animals) and fenbendazole 50 mg/kg orally once daily for 5 days. All animals completed the last dose of this treatment > 8 weeks prior to challenge except CJF2, BF61, 4399, 4376, 4402, 4470 which completed the treatment less than 4 weeks prior to challenge. Due to impacts of NCI facility renovations, some animals were not maintained under barrier husbandry conditions employed as described for the ONPRC study for the entirety of this period. Additionally, 3 animals (4550, 4543, DEJ8) also received cephalosporin treatments for wounds after receiving the prophylaxis treatment regimen but prior to intrarectal (IR) challenge and KNL, DEZ2, and DEJ8 also received a second 5-day course of fenbendazole approximately one month after the initial treatment. Six animals (4470, 4402, 4376, 4399, BF61, and CJF2) were challenged with the same 3x10^3^ IU dose of SIVmac239X followed by serial euthanasia occurring on 3-, 5-, or 7- days post infection to assess early transmission dynamics. Following necropsy, multiple tissues, including multiple samples from the site of exposure, were screened for viral RNA and DNA by qRT-PCR, as previously described (Deleage et al. 2019, newly added reference, see [2] in the reference list for this notice). With no viral nucleic acid detected, these animals were considered uninfected. We therefore increased the challenge dose to 3x10^4^ and 4 animals were intrarectally challenged (4550 and 4543) or rechallenged (4317 and 4324). Two animals (4317 and 4324) were again determined to be uninfected following qRT-PCR assessment of blood plasma. One animal (4550) was qRT-PCR negative in blood and relevant tissues at the d14 necropsy while the other (4543) was qRT-PCR positive in both blood and multiple tissues at the d14 necropsy. Due to the variable outcome at the 3x10^4^ IU dose, two animals were challenged (DEJ8 and 4397) and four others rechallenged (4317, 4324, DEZ2, and KNL) with 3x10^5^ IU of SIVmac239X. All 6 animals became productively infected with normal plasma viral load kinetics and plasma from each animal containing between 1–4 founder variants based on viral sequencing.

Data from the NCI study are presented in Table 1 of [1]. The authors provide a revised Table 1 which has been updated to include details of any variations in treatment for each animal (see Table 1 footnotes).

**Table 1:** Evaluation of acquisition of intrarectal challenged SIVmac239X and enumeration of transmitted/found variants in multimodal treated and untreated rhesus macaques.

Multimodal therapy naive				
Animal	Dose	Infection	T/F counts	AID_50_ ~1,000 IU
ZD47	1,000 IU	No	0	
DD36	1,000 IU	Yes	2	
			
ZD49	3,000 IU	Yes	6	
ZD76	3,000 IU	Yes	8	
Multimodal therapy treated				
Animal	Dose	Infection	T/F counts	
4470^*^	3,000 IU	No	0	AID_50_ ~100,000 IU
4402^*^	3,000 IU	No	0	
4376^*^	3,000 IU	No	0	
4399^*^	3,000 IU	No	0	
BF61^*^	3,000 IU	No	0	
CJ2F^*^	3,000 IU	No	0	
4550^^^	30,000 IU	No	0	
4317	30,000 IU	No	0	
4324	30,000 IU	No	0	
4543^^^	30,000 IU	Yes	2	
DEJ8^^^^#^	300,000 IU	Yes	1	
4397	300,000 IU	Yes	2	
DEZ2^#^	300,000 IU	Yes	2	
KNL^#^	300,000 IU	Yes	2	
4317	300,000 IU	Yes	2	
4324	300,000 IU	Yes	4	

*Completed treatment less than 4 weeks prior to challenge

^Received cephalosporin for wounds prior to challenge

#Received second course of fenbendazole ~1 month after initial treatment

All other parts of the article (Table 2, Figs 1–10 and S1-S5) report data from the follow-up ONPRC study only. There are additional instances where the language describing treatment in the NCI animals is incorrect in the main text of the manuscript. These errors are corrected here:

In the fifth sentence of the Author Summary, “GPF” is incorrectly used instead of “treated” to describe the NCI study animals. The correct sentence is, “Finally, compared to treatment-naïve controls, treated animals challenged with SIV intrarectally demonstrated a more controlled and consistent rate of SIV acquisition, suggesting underlying EPs even at subclinical levels maybe associated with confounding variability between study subjects”.

There are errors in the description of the components of the multimodal treatments, as follows:

- In the second sentence of the third paragraph of the Introduction, the correct sentence is “There was an ~100 fold increase in intrarectal (IR) SIVmac239X dose required to infect 50% of the animals (AID50) receiving treatment for common GI pathogens utilizing a short course multimodal therapeutic regimen (metronidazole and fenbendazole), compared to untreated animals”.
- In the fourth sentence of the first paragraph in the Results section, the correct sentence is “Of six animals that received multimodal treatment immediately before challenge (metronidazole and fenbendazole), none had detectable virus when identically challenged with an inoculum dose of 3,000 IU”.

Finally, for clarity, the following sentence should be omitted from the Discussion because it is speculative, referring to indirect evidence, while the animals included in the NCI study were not tested sufficiently to confirm microbiome status prior to or after treatment: “Other animals from the same shipment/source as the proof-of-concept animals had positive results for Shigella spp., Strongyloides spp., Balantidium spp. and Entamoeba spp., prior to treatment and post-treatment, for the cohort that received the short course multimodal therapy, the proof of concept animals were housed in a facility which excluded Shigella spp., and parasitic EPs (Entamoeba spp., Balantidium spp., Strongyloides spp., and Trichuris spp.), and were longitudinally confirmed free of EPs through serial diagnostic sampling.”

As acknowledged in the article [1], the regimens used in the NCI study varied relative to that used in the ONPRC study owing to the differing common background pathogens at the two facilities, though all animals received treatment targeted to reduce GI pathogen burden. The authors do not believe these differences affect the results, conclusions or understanding of the paper, which are fundamentally based on the data generated from the ONPRC study.

The initial dose titration study at NCI demonstrated that treatment for common endemic pathogens correlated with reduced rectal acquisition risk for SIV, but the NCI study design does not provide mechanistic insight for a causal link. The ONPRC study looked at probable mechanisms for the reduced rectal SIV acquisition observed in the NCI study, focusing on improvements in mucosal barrier integrity and reduced background inflammation in GI pathogen-free (GPF) animals, but did not formally test SIV acquisition in these animals. Therefore, while changes to mucosal barrier integrity and local and systemic inflammation may be expected to reduce acquisition risk for rectal SIV based on what is known, unequivocal conclusions cannot be drawn from the studies reported in [1] about a causal link between the multimodal treatment regimens tested and rectal SIV acquisition risk.

### Assessment of the multimodal treatment regimen at ONPRC

The authors provide the following corrections and clarifications regarding the assessment of the multimodal treatment regimen (ONPRC study):

The second sentence of the last paragraph of the Introduction is corrected to state “We also demonstrated that the regimen and associated practices were capable of creating and maintaining GPF status in rhesus macaques for 6 months.”

In the fifth sentence of the third paragraph of the Results section, the correct sentence is “The choice to use enrofloxacin, paromomycin, and fenbendazole concurrently was based on the bactericidal activity of these drugs and prior experience eliminating the common pathogens (except Campylobacter spp.) using this combined regimen.”

The third sentence of the first paragraph of the Discussion section is corrected to state “As the vast majority of animals screened did not meet these selection criteria, the animals presented in this work represent a healthy cohort of animals at the ONPRC and likely minimized the potential to demonstrate a positive impact of the therapy.”

The fifteenth sentence of the third paragraph of the Discussion section is clarified with updated wording as follows: “Antibiotic resistance could become an issue, however several factors help to mitigate this risk; 1) to date we have not detected any of the pathogenic bacteria we are attempting to eliminate after completion of the therapeutic regimen as evidenced by the 3 negative tests required for achieving GPF status with all animals where GPF status has been sought successfully achieving GPF status, 2) though an in-depth assessment has not been performed, we have not observed increased rates of antibiotic resistance in other bacterial populations in treated animals when clinical samples are taken for procedures such as wound care, though the number of samples to date in treated cohorts is quite small, and 3) as these models should be terminal, and given the practices employed, there would be little risk of transmitting resistant bacteria to other animals/cohorts if they were to occur.”

The second sentence of the Materials and Methods section titled “Assessment of the multimodal treatment regimen” is corrected to state "All animals were captive-born at ONPRC and inhabited socially-housed indoor/outdoor enclosures before their transfer to indoor standardized housing prior to study start and housed for the duration of the study in accordance with the IACUC protocol and facility practices."

Two animals included in the assessment of the multimodal treatment regimen (ONPRC study) did not fully meet the pre-study criteria reported in the article; however, the authors consider that this did not impact the study. In the third sentence of the Materials and Methods section titled Assessment of the multimodal treatment regimen, the correct sentence is “All enrolled macaques were research naïve, had not received antibiotics for at least 2 months, had no GI disease requiring antibiotics for at least 6 months, and were SPF (serologically negative for simian T-lymphotropic virus-1, SIV, simian type D retrovirus, and Macacine herpes 1) prior to the start of the study, with two exceptions: animal RM14 received azithromycin for diarrhea 5 months and 5 days prior to the start of the study, and animal RM6 received cephalexin to treat a wound 1 month and 19 days prior to study start.”

The term “Research Naïve” in the above sentence indicates no prior manipulations occurred which would be expected to impact research outcomes, however research macaques often have some assessments, such as behavior, blood and other minimally invasive sampling prior to research use.

The fourth section heading in the Materials and Methods is clarified with updated wording as follows: Description of standard practices to maintain gastrointestinal pathogen free (GPF) status in macaque colonies.

The authors also provide the following additional information regarding the 16-RM study conducted at ONPRC to assess the multimodal treatment regimen:

Five macaques that were initially included in the ONPRC study were replaced because they were unwittingly treated with probiotics after the first microbiome samples were taken. All of the data reported in [1] represents the results from 16 macaques, a group that included replacements for the animals that received probiotics. These 16 macaques were followed for 5.5–6 months post-treatment and were not exposed to probiotics or antibiotics post study treatment as detailed in the paper permitting unconfounded assessment of the microbiome impacts of the study regimen. The published article [1] did not use any data from the five animals that were removed from the study; the exclusion of these animals did not impact the study.

The individual-level quantitative data underlying all charts in the article are provided here as S1 File.

## Supporting information

S1 File Underlying Data. The quantitative data underlying Figs 4–10 and Fig S1.
(XLSX)
