# Supplementary material for: Mitigation of endemic GI-tract pathogen-mediated inflammation through development of multimodal treatment regimen and its impact on SIV acquisition in rhesus macaques
Source: PLoS Pathog. 2021 May 10;17(5):e1009565. doi: 10.1371/journal.ppat.1009565 (PMC8148316; doi:10.1371/journal.ppat.1009565)
Supplement: S1 Fig — (A) Representative images of formaldehyde-fixed, paraffin-embedded descending colon biopsies demonstrate the dramatically reduced neutrophil recruitment (MPO) observed following the clean-up regimen. (B) IHC quantification of CD4+ T cells and macrophages (CD68/CD163) was performed using HALO, which demonstrated markedly reduced inter-animal variance (P = 0.0187) assessed by Wilcoxon signed-rank test. (C) Despite a negligible change in overall IHC staining (CD4+ and CD68/CD168) between pre and post-treatment groups (scale bars = 100μm). (PDF) [file ppat.1009565.s001.pdf]

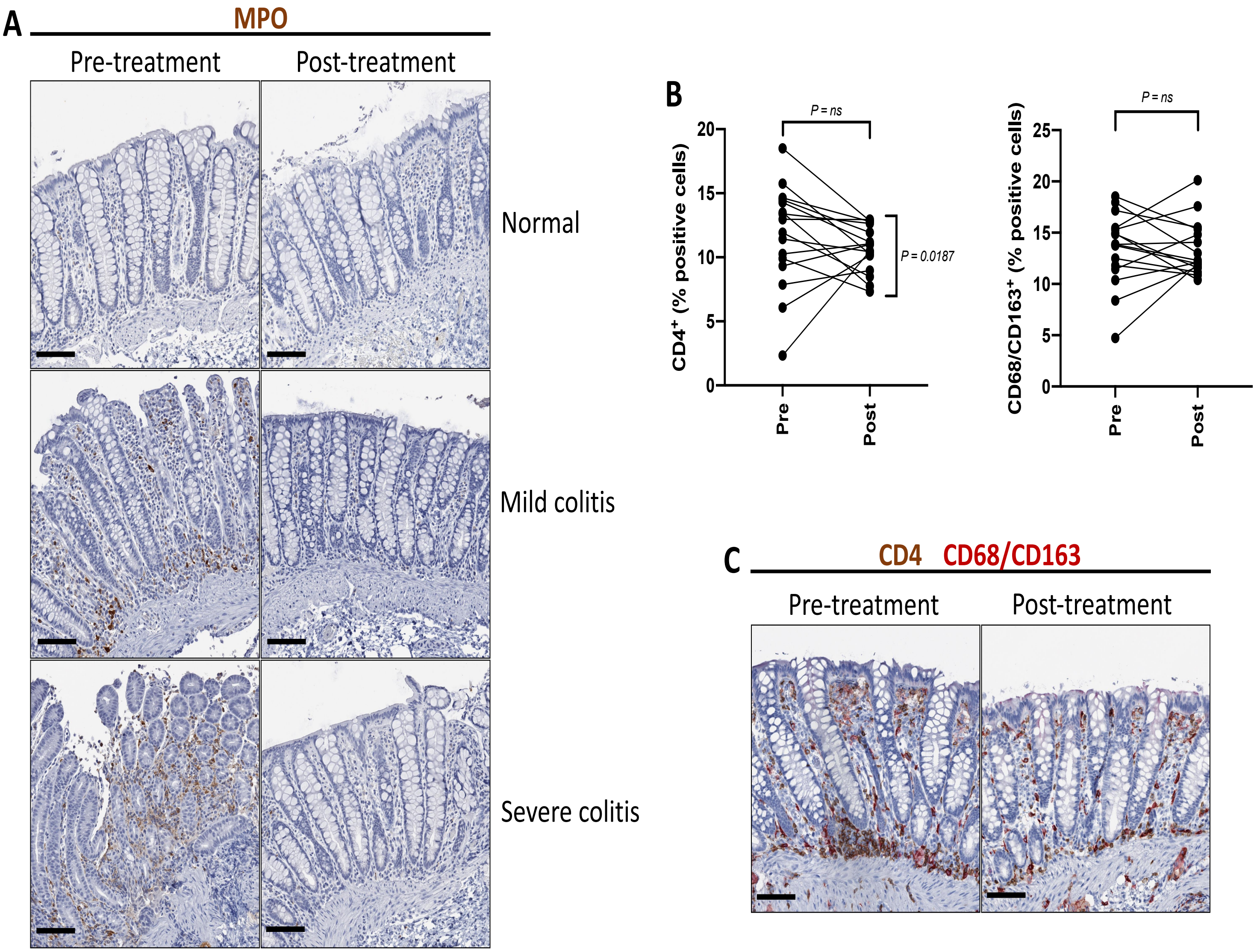

Supplemental Figure 1. Inflammation is reduced, and T cell infiltration normalized, in the descending colon following treatment.

(A) Representative images of formaldehyde-fixed, paraffin-embedded descending colon biopsies demonstrate the dramatically reduced neutrophil recruitment (MPO) observed following the clean-up regimen. **(B)** IHC quantification of CD4+ T cells and macrophages (CD68/CD163) was performed using HALO, which demonstrated markedly reduced inter-animal variance (P=0.0187) assessed by Wilcoxon signed-rank test. **(C)** Despite a negligible change in overall IHC staining (CD4+ and CD68/CD168) between pre and post-treatment groups (scale bars = 100μm).
