# Supplementary material for: Mitigation of endemic GI-tract pathogen-mediated inflammation through development of multimodal treatment regimen and its impact on SIV acquisition in rhesus macaques
Source: PLoS Pathog. 2021 May 10;17(5):e1009565. doi: 10.1371/journal.ppat.1009565 (PMC8148316; doi:10.1371/journal.ppat.1009565)
Supplement: S2 Fig — Representative images of Mx1, pSTAT1, and H&E-stained sections of colon exhibiting the pre-and post-treatment changes observed with treatment designed to create GPF status. Pre-treatment sections (left-hand column) had higher staining for (A) Mx1, (B) pSTAT1, as well as (C) increased severity of inflammation and spread to the submucosa (asterisk), increased epithelial hyperplasia and necrosis (arrowhead), and more superficial macrophages with intracytoplasmic cellular debris. Some high-scoring sections had crypt abscesses (arrow). Post-treatment, minimal (A) Mx1 and (B) pSTAT1 were observed, and (C) low-scoring sections were relatively quiescent with normal submucosal gut-associated lymphoid tissue (dotted line) and low numbers of lamina propria lymphocytes. (PDF) [file ppat.1009565.s002.pdf]

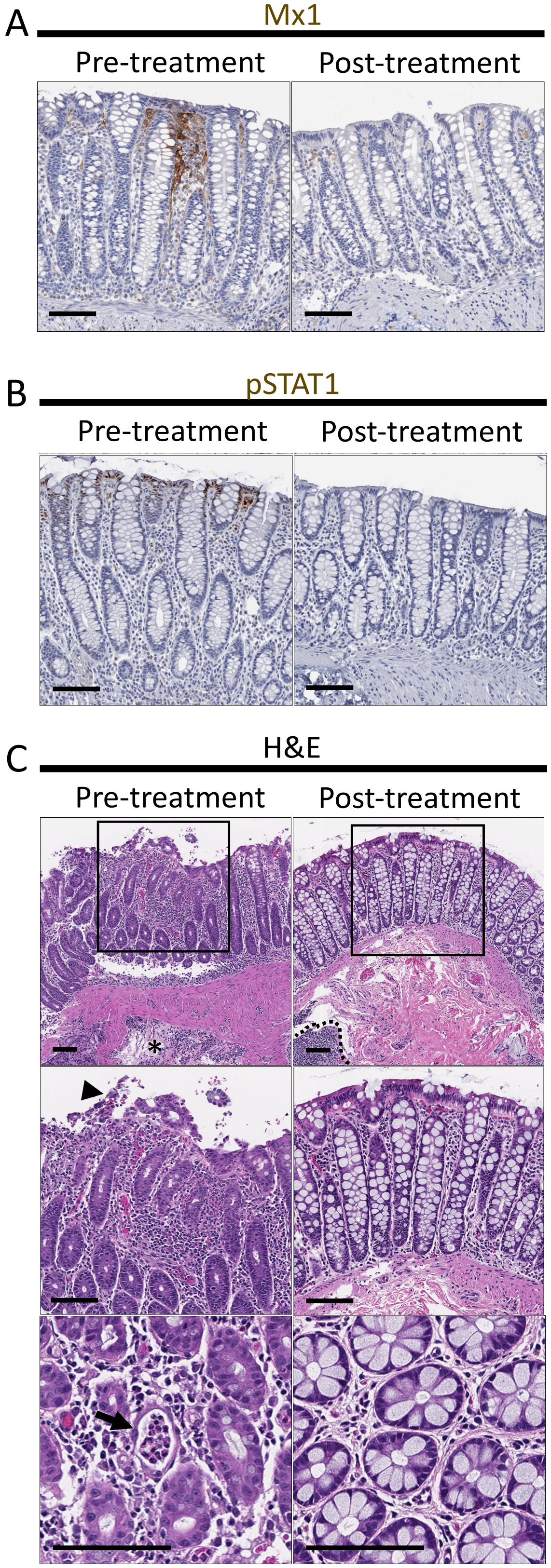

Supplemental Figure 2. IHC and histological measures of colonic inflammation significantly reduced following treatment.

Representative images of Mx1, pSTAT1, and H&E-stained sections of colon exhibiting the pre- and post-treatment changes observed with treatment designed to create GPF status. Pre-treatment sections (left-hand column) had higher staining for **(A)** Mx1, **(B)** pSTAT1, as well as **(C)** increased severity of inflammation and spread to the submucosa (**asterisk**), increased epithelial hyperplasia and necrosis **(arrowhead)**, and more superficial macrophages with intracytoplasmic cellular debris. Some high-scoring sections had crypt abscesses **(arrow)**. Post-treatment, minimal **(A)** Mx1 and **(B)** pSTAT1 were observed, and **(C)** low-scoring sections were relatively quiescent with normal submucosal gut-associated lymphoid tissue **(dotted line)** and low numbers of lamina propria lymphocytes.
