# Supplementary material for: Mitigation of endemic GI-tract pathogen-mediated inflammation through development of multimodal treatment regimen and its impact on SIV acquisition in rhesus macaques
Source: PLoS Pathog. 2021 May 10;17(5):e1009565. doi: 10.1371/journal.ppat.1009565 (PMC8148316; doi:10.1371/journal.ppat.1009565)
Supplement: S3 Fig — Whole blood and tissue-derived lymphocytes were stained using fluorophore conjugated monoclonal antibodies and analysis was performed using a LSR II Becton Dickinson instrument. The resulting list mode multi-parameter data files were analyzed using the FlowJo software program (version 9.9.6; Ashland, OR: Becton, Dickinson and Company; 2020) with the hierarchy shown here. CD3+ small lymphocytes were selected and divided into CD4+ and CD8+ T cell subsets, then defined as memory and naïve T cell subset populations based on CD28 and CD95 expression patterns. Memory subsets were then further differentiated by their expressions of CD28 and CCR7. Naïve (CD28+ CD95-), central memory (CD28+ CD95+ CCR7+), transitional memory (CD28+ CD95+ CCR7-), and effector memory (CD28- CD95+ CCR7-) T cells were then analyzed for subsets manifesting upregulation of the markers for activation (CD38) and proliferation (Ki67). (PDF) [file ppat.1009565.s003.pdf]

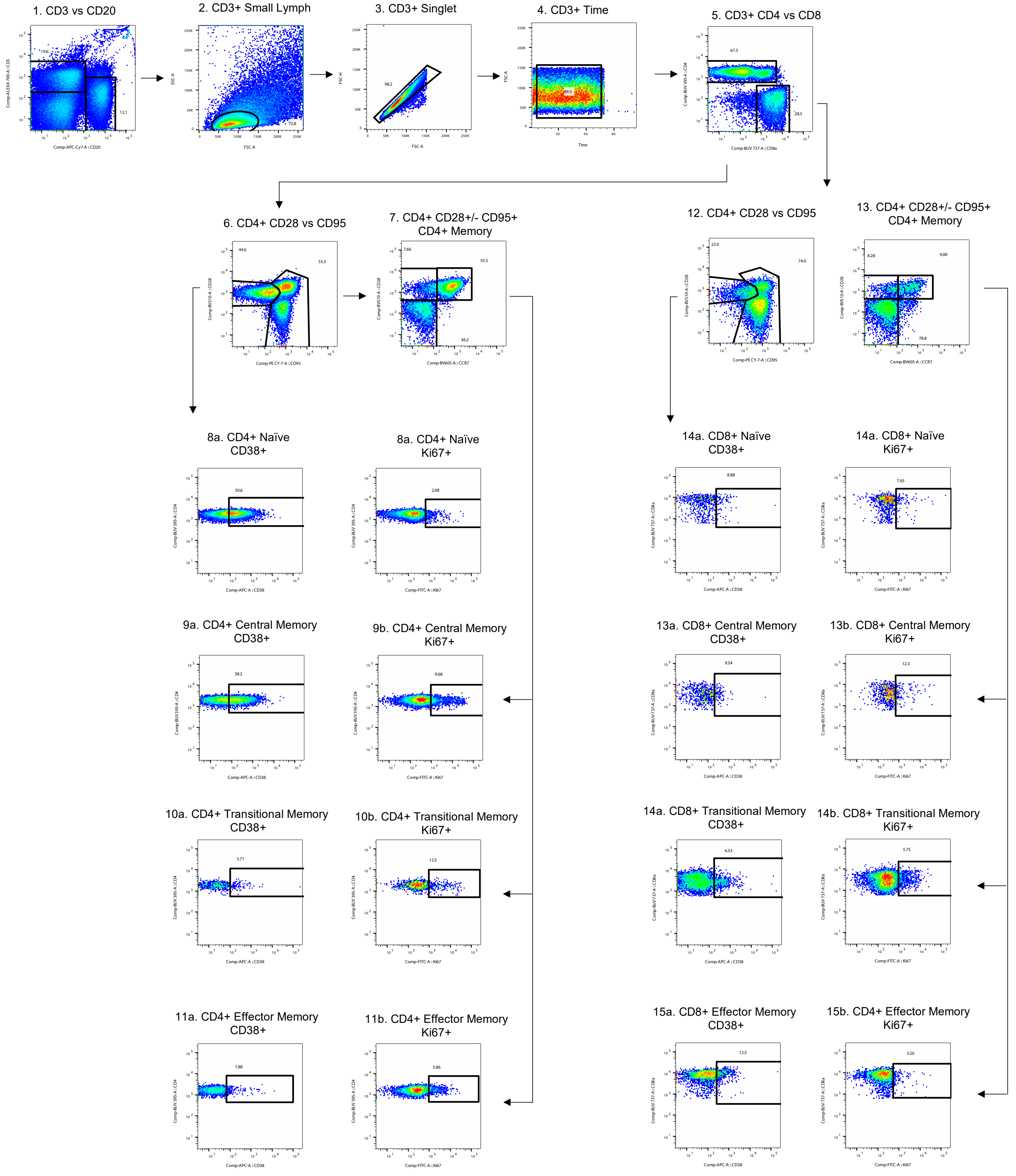

Supplemental Figure 3. T cell gated hierarchy.

Whole blood and tissue-derived lymphocytes were stained using fluorophore conjugated monoclonal antibodies and analysis was performed using a LSR II Becton Dickinson instrument. The resulting list mode multi-parameter data files were analyzed using the FlowJo software program (version 9.9.6; Ashland, OR: Becton, Dickinson and Company; 2020) with the hierarchy shown here. $CD3^+$ small lymphocytes were selected and divided into $CD4^+$ and $CD8^+$ T cell subsets, then defined as memory and naïve T cell subset populations based on CD28 and CD95 expression patterns. Memory subsets were then further differentiated by their expressions of CD28 and CCR7. Naïve ($CD28^+$ $CD95^-$), central memory ($CD28^+$ $CD95^+$ $CCR7^+$), transitional memory ($CD28^+$ $CD95^+$ $CCR7^-$), and effector memory ($CD28^-$ $CD95^+$ $CCR7^-$) T cells were then analyzed for subsets manifesting upregulation of the markers for activation (CD38) and proliferation (Ki67).
