# Supplementary material for: Mitigation of endemic GI-tract pathogen-mediated inflammation through development of multimodal treatment regimen and its impact on SIV acquisition in rhesus macaques
Source: PLoS Pathog. 2021 May 10;17(5):e1009565. doi: 10.1371/journal.ppat.1009565 (PMC8148316; doi:10.1371/journal.ppat.1009565)
Supplement: S4 Fig — Whole blood and tissue-derived lymphocytes were stained and analyzed using the software described above. CD20+ small lymphocytes were selected and divided into memory (IgD+/- CD27+) and naïve (IgD+ CD27-) B cell subsets. The subsets were further analyzed for upregulation of activation marker CD38, proliferation marker Ki67, complement receptor CD21, as well as surface expression of immunoglobulins G and M. (PDF) [file ppat.1009565.s004.pdf]

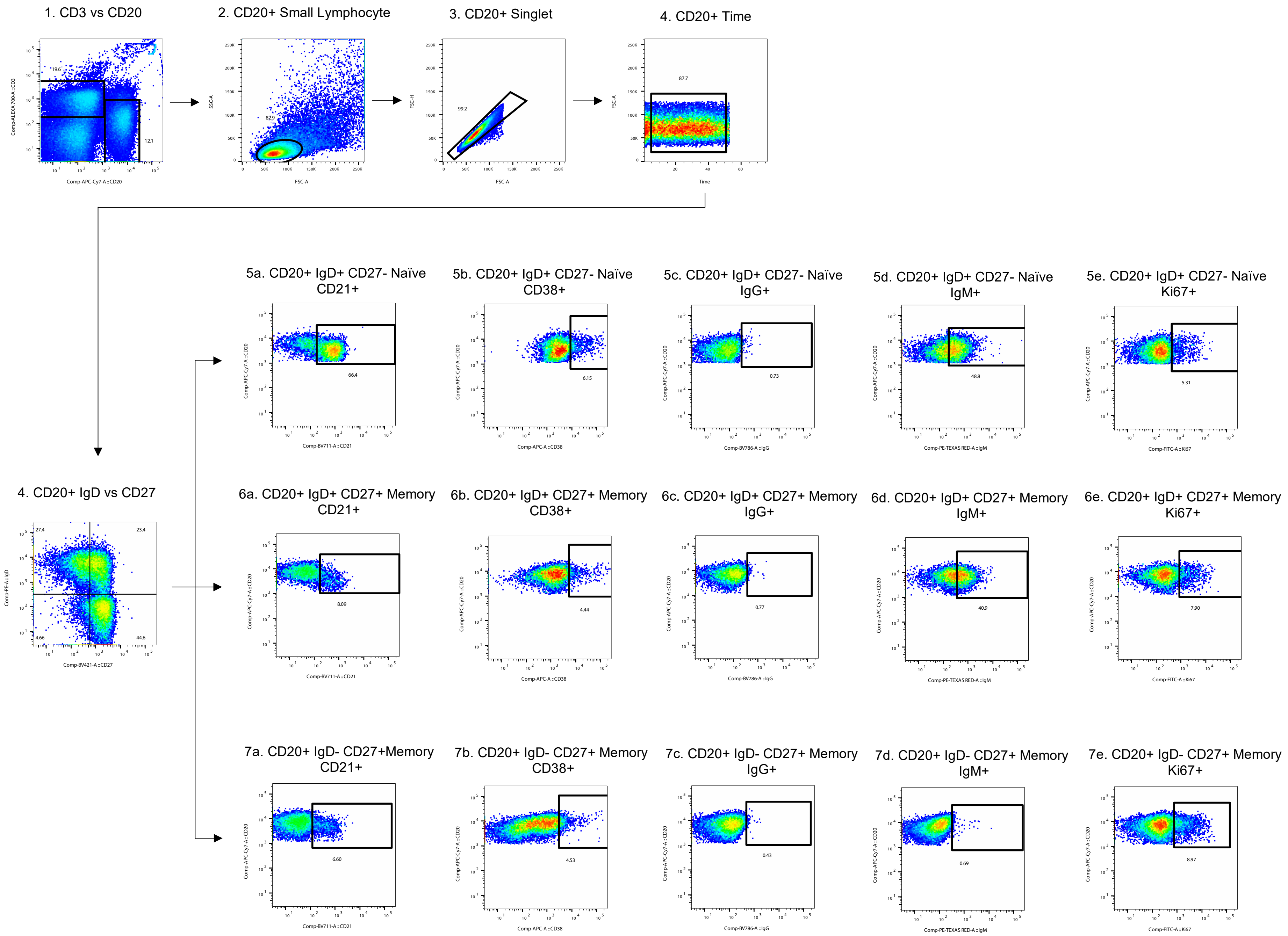

Supplemental Figure 4. B cell gated hierarchy.

Whole blood and tissue-derived lymphocytes were stained and analyzed using the software described above. $CD20^{+}$ small lymphocytes were selected and divided into memory ($IgD^{+/-}$ $CD27^{+}$) and naïve (IgD^{+} $CD27^{-}$) B cell subsets. The subsets were further analyzed for upregulation of activation marker CD38, proliferation marker Ki67, complement receptor CD21, as well as surface expression of immunoglobulins G and M.
