# Supplementary material for: Mitigation of endemic GI-tract pathogen-mediated inflammation through development of multimodal treatment regimen and its impact on SIV acquisition in rhesus macaques
Source: PLoS Pathog. 2021 May 10;17(5):e1009565. doi: 10.1371/journal.ppat.1009565 (PMC8148316; doi:10.1371/journal.ppat.1009565)
Supplement: S5 Fig — Whole blood and tissue-derived lymphocytes were stained and analyzed using the software described above. CD3- CD20- HLA-DR+ lymphocytes were selected and divided into monocyte populations based on CD14 and CD16 expression. Dendritic cells were further defined by their lack of CD14 and CD16 as well as their expression of CD11c (myeloid DC) and CD123 (plasmacytoid DC). Upregulation of the marker CD169 was also measured on all HLA-DR+ lymphocytes. (PDF) [file ppat.1009565.s005.pdf]

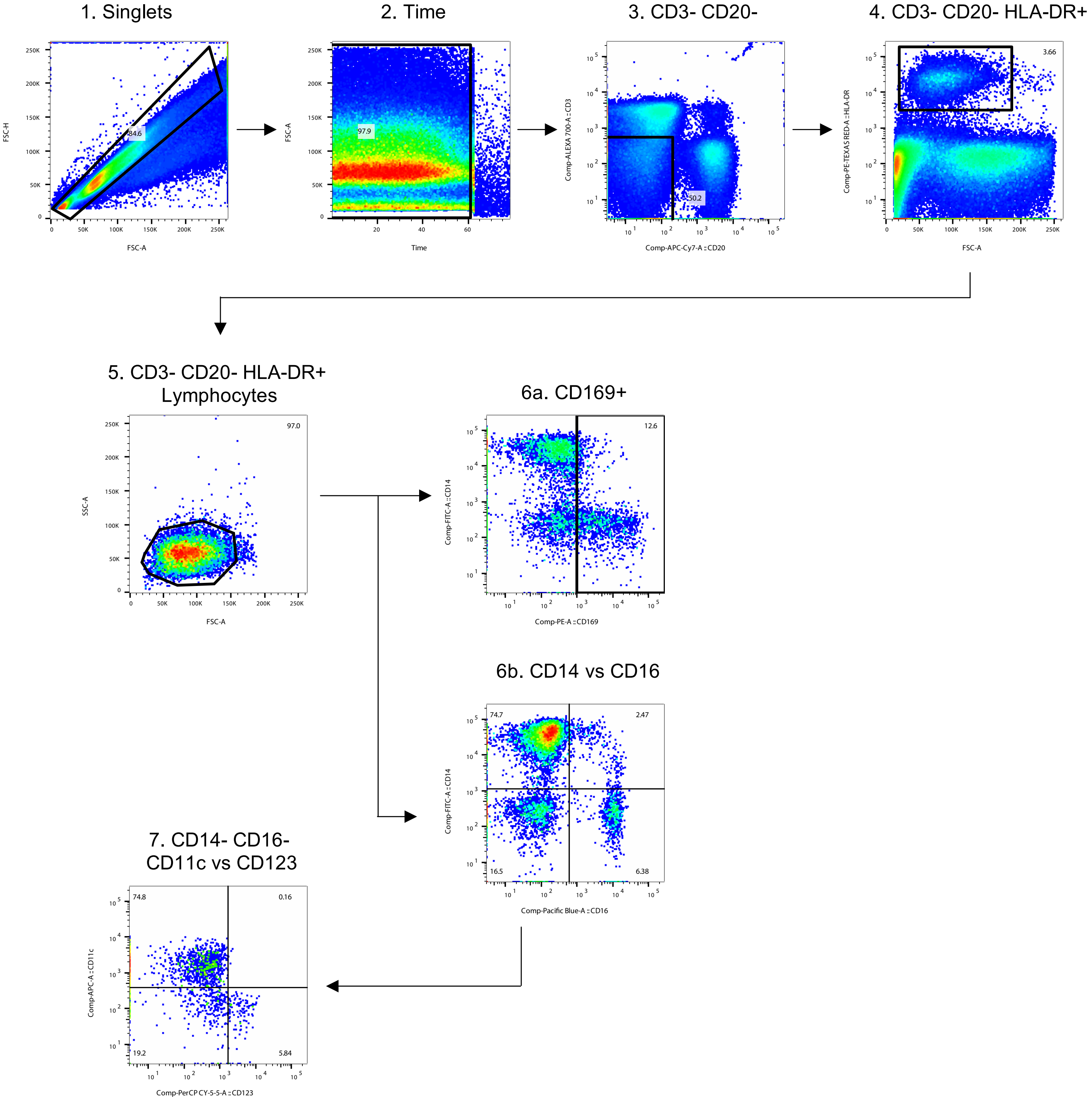

Supplemental Figure 5. Dendritic cell, monocyte/macrophage hierarchy.

Whole blood and tissue-derived lymphocytes were stained and analyzed using the software described above. $CD3^-$ $CD20^-$ HLA-DR^+ lymphocytes were selected and divided into monocyte populations based on CD14 and CD16 expression. Dendritic cells were further defined by their lack of CD14 and CD16 as well as their expression of CD11c (myeloid DC) and CD123 (plasmacytoid DC). Upregulation of the marker CD169 was also measured on all HLA-DR^+ lymphocytes.
